# Quantifying the Frictional Forces between Skin and Nonwoven Fabrics

**DOI:** 10.3389/fphys.2017.00107

**Published:** 2017-03-06

**Authors:** Kavinda Jayawardana, Nicholas C. Ovenden, Alan Cottenden

**Affiliations:** ^1^Department of Mathematics, University College London (UCL)London, UK; ^2^Department of Bioengineering and Medical Physics, University College London (UCL)London, UK

**Keywords:** friction, nonwoven fabrics, shell theory, capstan equation, abrasion dermatitis, incontinence, free boundary

## Abstract

When a compliant sheet of material is dragged over a curved surface of a body, the frictional forces generated can be many times greater than they would be for a planar interface. This phenomenon is known to contribute to the abrasion damage to skin often suffered by wearers of incontinence pads and bed/chairbound people susceptible to pressure sores. Experiments that attempt to quantify these forces often use a simple capstan-type equation to obtain a characteristic coefficient of friction. In general, the capstan approach assumes the ratio of applied tensions depends only on the arc of contact and the coefficient of friction, and ignores other geometric and physical considerations; this approach makes it straightforward to obtain explicitly a coefficient of friction from the tensions measured. In this paper, two mathematical models are presented that compute the material displacements and surface forces generated by, firstly, a membrane under tension in moving contact with a rigid obstacle and, secondly, a shell-membrane under tension in contact with a deformable substrate. The results show that, while the use of a capstan equation remains fairly robust in some cases, effects such as the curvature and flaccidness of the underlying body, and the mass density of the fabric can lead to significant variations in stresses generated in the contact region. Thus, the coefficient of friction determined by a capstan model may not be an accurate reflection of the true frictional behavior of the contact region.

## 1. Introduction

Pressure ulcers are an area of localized cutaneous damage typically associated with pressure from bony protuberances on aged skin. They can develop when a large amount of pressure is applied to an area of skin over a short period of time or occur when less pressure is applied over a prolong period of time. When pressure is applied to soft tissue, it may result in completely or partially obstructed blood flow to the soft tissue, starving the tissue of oxygen and nutrients, which eventually leads to necrosis in the affected area, and thus an ulcer. Shear (i.e., constant and prolonged static friction) is also a cause, as it can pull on blood vessels that feed the skin, consequently restricting the blood flow. Pressure ulcers often occur in very sedentary individuals, such as those with impaired mobility (Maklebust and Sieggreen, 2001).

It is assumed that friction contributes to skin damage via stripping of the epidermal layer of the skin, creating an environment conducive to further skin damage due to friction. An alteration in the coefficient of friction increases the skin's adherence to the outside surface, which can eventually lead to wounds and infections. A publication by Murray et al. (2001) highlights many preventive measures for pressure ulcers. For example, to eliminate shear and friction, it is recommended that the exposed skin is covered by protective dressings, padding or sheepskin. For those who are bedridden, elevating the foot of bed to 20° is advised when sitting to prevent sliding as well as maintaining the head of the bed at the lowest possible elevation, consistent with the individual's medical condition and comfort.

Athletes, due to excessive movement, are also subject to repeated mechanical trauma to the skin, which is often painful (Bergfeld and Taylor, 1985). Conditions such as fissure of the nipple (Powell, 1983; Conklin, 1990) and friction blisters (Herring and Richie, 1990) are well documented traumas to the skin caused by friction. It is also established that friction plays a role in the development of dermatitis in other settings. In many cases, it is observed that friction damages the stratum corneum and the stratum basale to varying degrees (Wilkinson, 1985). Friction is also believed to play a significant role in incontinence-associated dermatitis with published literature on the coefficient of friction (measuring techniques and actual measurements) between human skin and fabrics in incontinence related publications (Berg, 1987; Cottenden et al., 2008a,b; Cottenden and Cottenden, 2009). The NHS estimates that between 3 and 6 million adults in the UK have some degree of urinary incontinence (Irwin et al., 2006), and the prevalence is set to increase due to an aging population. It is documented that the wearing of incontinence pads over prolong periods of time is a major cause of incontinence associated dermatitis. Although the pads absorb moisture, they can also act as a barrier that prevents water from escaping. This leads to over-hydration of the stratum corneum in the epidermis (i.e., the upper most part of the skin). Scheuplein and Blank (1971) found that an increase in skin hydration leads to an increase in the thickness of the stratum corneum, resulting in a weakening of the cell structure. Moreover, tests conducted on adults and on infants showed that over-hydration of the stratum corneum is responsible for a threefold increase in the coefficient of friction.

It is estimated that over 400, 000 individuals develop a new pressure ulcer annually in the UK (mainly the elderly) and approximately 51, 000 of them will be admitted to hospital (Farage et al., 2007). A study conducted in 1993 showed that the cost to the NHS of treating pressure ulcers was around £180 − £321 million, approximately 0.4–0.8% of total health spending (Touche, 1993). However, more recently this figure was considered to be a substantial underestimate, even allowing for inflation (Bennett et al., 2004). Bennett et al. (2004) found that the cost of treating pressure ulcers in UK (excluding methicillin-resistant Staphylococcus aureus (MRSA), surgical interventions and litigation costs) ranges between £1.4 to £2.1 billion annually, which is over 4% of gross NHS expenditure. The costs were deduced by estimating the daily cost of the resources required to deliver protocols of care reflecting good clinical practice. In the USA, the Omnibus Budget Reconciliation Act in 1987 made it easier for claimants to prove that a provider had been negligent following the development of pressure ulcers. Between 1992 and 1996, the median settlement value following successful litigation for negligence regarding a pressure ulcer was $279,000 (Thomson and Brooks, 1999).

The above evidence strongly indicates that preventive measures are immensely important. Lowering the prevalence of pressure ulcers should lead to fewer resources being spent their treatment, thus reducing overall spending in the NHS. To design new products and protocols that reduce skin damage, there has been a great deal of experimental work aiming to accurately quantify the mechanical forces generated by fabrics and other materials in contact with human skin and its response (Dowson, 1996; Sivamani et al., 2003; Silver et al., 2003; Cottenden et al., 2008b). In these experiments it is vital to quantify the relationship between normal and tangential forces across the contact region and this is often achieved by determining a coefficient of friction (Gwosdow et al., 1986; Zhang and Mak, 1999; Cottenden et al., 2008b).

**Figure 1:**
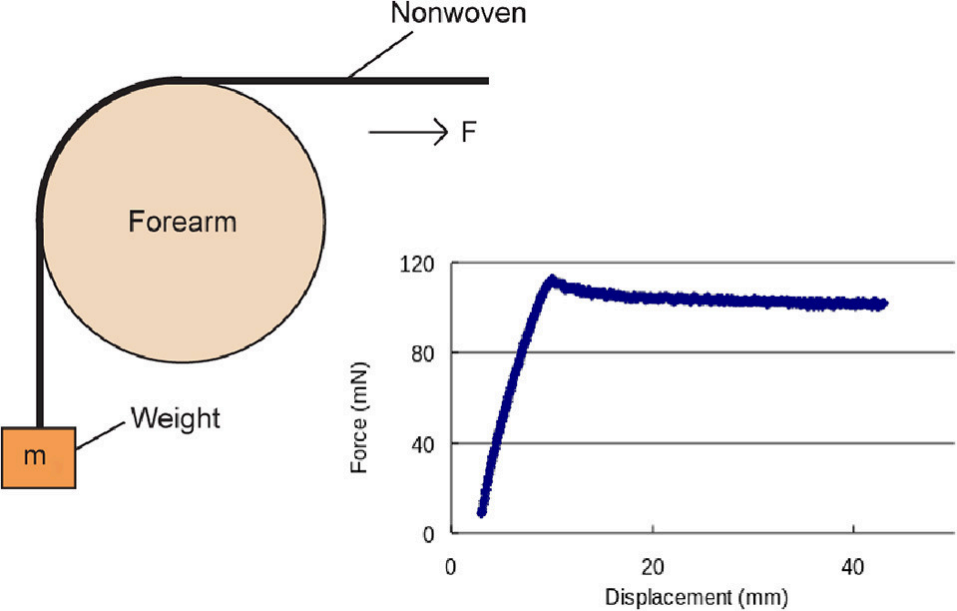
**Basic setup of experiments to determine the coefficients of friction and a plot of a typical tensometer trace from Cottenden et al. (2008a,b)**. Image courtesy of Dr. Sabrina Falloon, UCL.

In recent years, the UCL Continence & Skin Technology group has developed and validated a novel method for measuring friction between fabrics and skin (Cottenden et al., 2008a,b), as well as developing mathematical models (Cottenden and Cottenden, 2009) to determine coefficients of friction. One aim of this research is to predict the magnitude of potentially damaging frictional forces generated between skin in contact with certain fabrics, such as the nonwoven materials used in incontinence pads. Figure 1 shows the basic setup of the experiments and a typical force trace obtained by the tensometer measurements. The tensometer is used to measure the force needed to drag a strip of nonwoven fabric (30 mm wide in the work referenced here) over the volar forearm of a volunteer while a weight (an applied mass, *m*) is attached to the other end of the strip. The arm is supported such that its upper surface, in the vicinity of the fabric strip, is held horizontal and at the same height as the tensometer grips, while the other end of the strip hangs vertically in a plane perpendicular to the pull direction of the tensometer. The angle of the arc of contact between the arm and strip is typically π/2 although, in the group's work using arm phantoms (Cottenden et al., 2008a), angles other than π/2 were achieved by adjusting the height of the tensometer grips relative to the arm phantoms.

**Figure 2:**
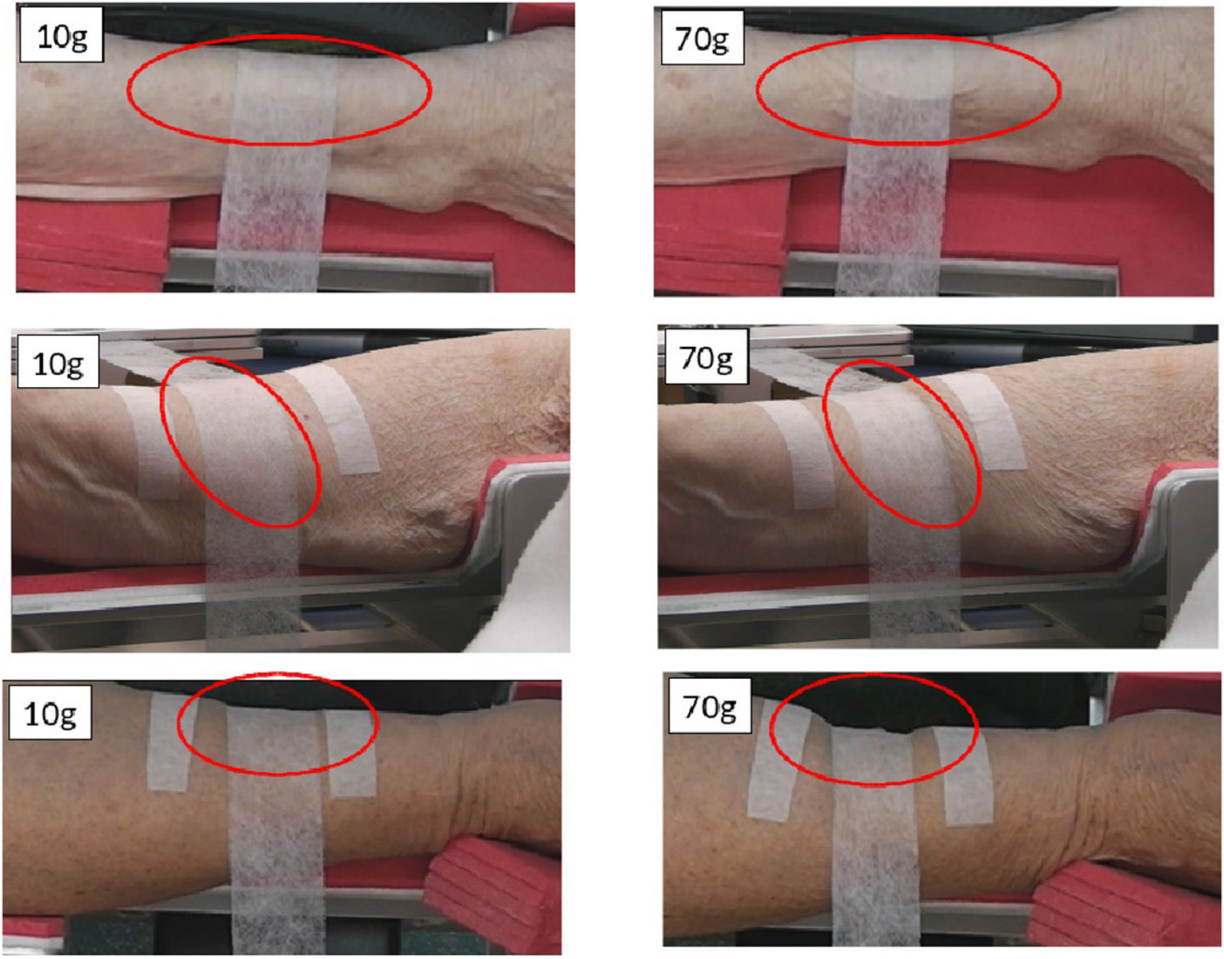
**Pictures of deformation in the skin and underlying soft tissue generated during measurements of friction between strips of nonwoven fabric and the volar forearms of female volunteers**. Forearms were held horizontal while a tensometer pulled nonwoven strips over the skin surface with an applied mass secured to the opposite (hanging) end of the fabric (10 and 70g). Images courtesy of Dr. Sabrina Falloon, UCL. The work was conducted with the approval of London Stanmore Research Ethics Committee and The Whittington Hospital NHS R&D office, September 2011.

One remarkable aspect of the experimental results obtained is that their rather simple capstan-type model (Rao et al., 2003; Jung et al., 2008), based only on the ratio of tensions at either end of the fabric strip and the arc of contact, appears able to produce reliable values for the coefficient of friction from experiments with various applied weights across different human subjects. This is despite the fact that during experiments significant skin deformation, rucking and wrinkling is often observed (Figure 2).

The capstan equation is the most perfect example of a belt-friction model, which describes behavior of a belt-like object moving over a rigid obstacle subjected to friction (Rao et al., 2003). Consider a membrane (i.e., a two-dimensional elastic body) with zero-Poisson's ratio or a *string* (i.e., a one-dimensional elastic body with arbitrary Poisson's ratio) over a *rough* rigid cylinder subject to appropriate boundary conditions such that the body in question is at *limiting equilibrium*, in other words it is at the point of slipping. This is a simple belt-friction problem and its properties are well-described by the capstan equation. However, we ask: what if our rigid contact body is no longer a cylinder, but some arbitrary geometry? Should a simple capstan equation apply to these geometries? Would a capstan-type equation still apply in the case where the underlying body is not rigid but *deformable*. Are the coefficients of friction obtained by a capstan equation reliable in such cases? These questions are the main focus of this paper and we examine the aspects of geometry and deformability of the underlying body separately in Sections 2 and 3 respectively.

In Section 2, we look at a thin membrane pulled dynamically at a constant speed over a rigid body. The section begins by introducing the capstan equation and highlighting some experimental data on arm phantoms that does not fit the predictions of the capstan model. We then extend an established model for Coulomb's law of static friction (Kikuchi and Oden, 1988) to curvilinear coordinates and use it in conjunction with a numerical model of a thin membrane pulled dynamically over a rigid body to investigate how the calculated coefficient of friction varies with (i) membrane parameters such as Poisson's ratio, Young's modulus and mass density; (ii) the speed and the applied tensions at the membrane edges; and (iii) the underlying geometry of the body, specifically, the Gaussian curvature. In Section 3, we examine the behavior of shells supported by elastic foundations when subjected to a friction condition. Faced with a free-boundary problem at the contact region, we use the same model for Coulomb's law of static friction (Kikuchi and Oden, 1988) to derive a more computationally tractable displacement-based static friction condition. We then take the overlying shell theory and use the displacement-based friction condition to transform the model into a constrained elastic two-body contact problem and explicitly derive the governing equations and the boundary conditions for the static friction problem of a thin shell-membrane on an elastic foundation. Finally, we present some numerical results to examine how the thickness and elasticity of the foundation affect the displacement and shear stress across the contact region. Discussion and conclusions follow in Section 4.

## 2. Modeling a non-woven fabric as a membrane supported by a rigid foundation with friction

### 2.1. Capstan equation and applications in friction modeling

The capstan equation or, as otherwise known, Euler's equation of tension transmission, is the relationship governing the maximum applied tension *T*_max_ with respect to the minimum applied tension *T*_0_ of an elastic string wound around a rough cylinder. The governing equation is given by

(1) $$T_{\text{max}}=T_{0}\exp(\mu_{F}\;\theta )\;,$$

where θ is the contact angle and μ_*F*_ is the coefficient of friction. By *string* we mean a one-dimensional elastic body and *rough* is an engineering term implying that the contact area exhibits friction. Note that the coefficient of friction is the physical ratio of the shear force and the normal force between two contacting bodies. In engineering, the capstan equation describes a body under a load, in equilibrium, involving friction between rope and a wheel-like circular object, and thus it is widely used to analyse the tension transmission behavior of cable-like bodies in contact with circular profiled surfaces (Jung et al., 2008; Baser and Konukseven, 2010) as well as in the field of robotics (Behzadipour and Khajepour, 2006).

As an example of the application of a capstan-type equation to experimental results, consider the results of Cottenden et al. (2008a) where the coefficient of friction is determined from experiments as that shown in Figure 1 of a non-woven fabric strip in contact with various arm phantoms made from Plaster of Paris and covered in Neoprene. Note, in particular, Figure 11 of Cottenden et al. (2008a) which shows the coefficients of friction obtained with different geometries, applied weights, and contact angles. While the capstan equation proves quite successful in obtaining coefficients of friction, the authors observe a steady increase in the mean coefficient of friction as the applied weight increases. Such dependence contradicts the assumptions of Equation (1). There is also variation in the coefficients of friction measured across different geometries and contact angles which seems to get wider as the applied mass is reduced. The authors acknowledge the apparent dependence of the coefficient of friction on the applied weight in Cottenden et al. (2008a) but highlight that the mean variation is small compared to the scatter of the data. The authors suggest that the departure from the capstan equation is likely to be caused by an interaction between the Neoprene on the underlying body and the moving nonwoven fabric at large tension.

**Figure 3:**
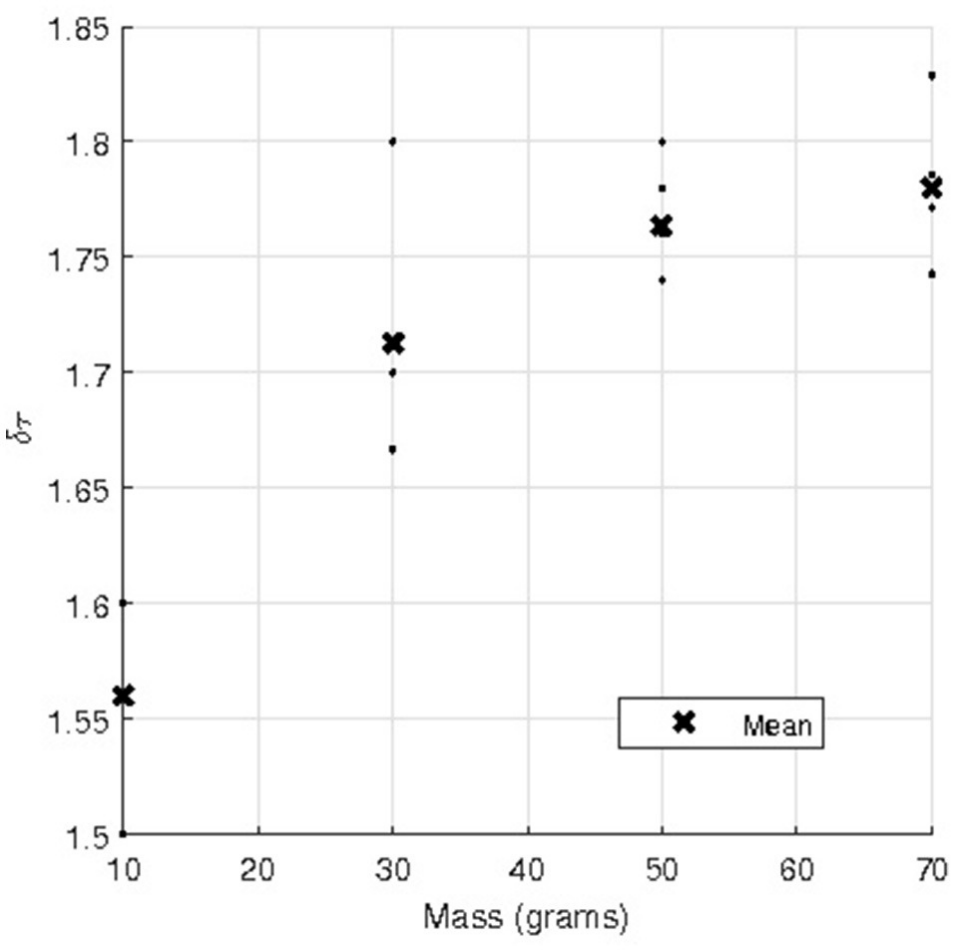
**Tension ratio against applied mass from the raw experimental data in Table 1 obtained from Karavokiros (2007)**.

The raw data of the case of a fabric strip in contact with an arm phantom of cylindrical cross section with a $\frac{127}{360}\pi$ contact angle can be found in Karavokiros' masters thesis (see table 2a of Karavokiros, 2007); for convenience, this raw data is reproduced here in Table 1. The table shows five repeated measurements involving dragging the fabric strip over the arm phantom with different applied masses (*m* in Figure 1). Figure 3 is a plot using these data of the measured tension ratio δτ = *T*_max_/*T*_0_, vs. the applied mass, where the mean ratio obtained for each applied mass is also shown. Note that Equation (1) implies that the tension ratio is constant for all applied masses, i.e., δτ = exp(μ_*d*_θ_0_), where μ_*d*_ and θ_0_ are constants. However, Figure 3 suggests as the applied mass increases, the tension ratio increases also. Of course, such an effect could be attributed to a number of factors, including experimental errors, but one possibility is that the standard capstan equation is simply not valid in these cases. To test this, we now develop two, more sophisticated, three-dimensional numerical models of a thin compliant sheet over an underlying body and vary the geometry and material properties of both the thin sheet and underlying body, as well as the applied tensions.

**Table 1:** **Tensometer readings: Plaster of Paris cylinder covered in Neoprene with $\frac{127}{360}\pi$ contact angle, where ***g*** is the acceleration due to gravity**.

**Applied Mass**	**Recorded Tension (*****F*****) 10**^**−3**^**N**					
**(*m*) in grams**	**Repeat Measurements**					**Mean**
10	16.0 g	15.0 g	15.0 g	15.0 g	16.0 g	15.6 g
30	51.0 g	54.0 g	51.0 g	50.0 g	51.0 g	51.4 g
50	88.0 g	87.0 g	89.0 g	87.0 g	90.0 g	88.2 g
70	125 g	124 g	128 g	122 g	124 g	125 g

*Applied mass m and tension F refer to the experimental schematic in Figure 1. Data courtesy of Karavokiros (2007)*.

### 2.2. Belt-friction model

In this section, we derive a pure-traction belt-friction model to describe the behavior of dynamic membranes supported by static rigid foundations.

Let ω ⊂ ℝ^2^ be a simply connected open bounded domain with the sufficiently smooth boundary ∂ω and let **σ** ∈ *C*^2^(ω; **E**^3^) be an injective immersion. Now assume that an isotropic elastic membrane is in contact with a rigid surface with positive mean curvature initially such that, in the stress-free configuration of the membrane, the contact area can be parameterized by the immersion **σ**(*x*^1^, *x*^2^) in curvilinear coordinates. Now we assert that the membrane is dynamic but the contact area remains static and constant as ω. Invoking a similar terminology to that of Cottenden and Cottenden (2009), the governing Cauchy momentum equations for the membrane stress τ^αβ^ (α, β = 1, 2) in tangential and normal directions can be derived thus

(2) $$\nabla_{\text{​}\alpha }\tau^{\alpha \beta }(u)+f_{r}^{\beta }(u)+g_{r}^{\beta }=\varrho \left(\partial_{tt}u^{\beta }+\Gamma_{\text{​}\alpha \gamma }^{\beta }\partial_{t}u^{\alpha }\partial_{t}u^{\gamma }\right)\;,$$

(3) $$F_{\text{​}[\text{II}]\alpha \gamma }\tau^{\alpha \gamma }(u)+f_{r}^{3}(u)+g_{r}^{3}=\varrho F_{\text{​}[\text{II}]\alpha \gamma }\partial_{t}u^{\alpha }\partial_{t}u^{\gamma }\;,$$

with the boundary conditions

(4) $$n_{\alpha }\tau^{\alpha \beta }(u){|}_{\partial \omega }=\tau_{0}^{\beta }{|}_{\partial \omega }\;,$$

where ***u*** ∈ *C*^2^(ω; ℝ^2^) is the displacement field and ϱ is the mass density of the membrane, $g_{r}\in C^{0}\left(\omega ;{\mathbb{R}}^{3}\right)$ is an external loading field, $f_{r}^{\beta }\left(u\right)$ are the shear force densities, $f_{r}^{3}\left(u\right)$ is the normal reaction density, ***n*** ∈ *C*^0^(ω; ℝ^2^) is the unit outward normal to the boundary and $\tau_{0}\in C^{0}\left(\omega ;{\mathbb{R}}^{2}\right)$ is the traction field applied to the boundaries of the membrane. The covariant *second fundamental form tensor* of **σ** with respect to the curvilinear coordinates is defined as

$$F_{\text{​}[\text{II}]\alpha \beta }=N_{i}\partial_{\alpha \beta }\sigma^{i}\;,\;\forall \;\alpha ,\beta \in \{1,2\}\;.$$

where $N=||\partial_{1}\sigma \times \partial_{2}\sigma |{|}^{-1}\left(\partial_{1}\sigma \times \partial_{2}\sigma \right)$ is the unit normal to the surface **σ**, × is the Euclidean cross product and || · || is the Euclidean norm.

Notice that this is a pure-traction problem. This implies that the boundary traction field **τ**_0_ cannot be arbitrary chosen. To proceed, we assume that the velocity, the acceleration and the force density fields are known and fixed prior to the problem. Typically here, in line with the tensometer experiments, we assume that the membrane moves at a given constant speed. The time-dependent translational part of the displacement is thus easily subtracted out, leaving the residual displacement *u*^β^. We now invoke the compatibility condition for pure-traction problems (see Section 1.3.4 of Necas et al., 2011 or Section 1.8 of Ciarlet, 2000) to find

(5) $$\begin{array}{l} \int_{\partial \omega }\tau_{0}^{\beta }w_{\beta }\;d(\partial \omega )\\ \;+\int_{\omega }\left(f_{r}^{\beta }(u)+g_{r}^{\beta }-\varrho \left(\partial_{tt}u^{\beta }+\Gamma_{\text{​}\alpha \gamma }^{\beta }\partial_{t}u^{\alpha }\partial_{t}u^{\gamma }\right)\right)w_{\beta }\;d\omega =0\;,\\ \;\forall \;w\in \{v\in H^{1}(\omega )|\epsilon (v)=0\}\;, \end{array}$$

where **ϵ**(·) is the strain tensor of a true-membrane. The compatibility condition implies that the internal forces are balanced by all the applied external forces.

Suppose now that the contact area is rough and the friction law governing this region is given by the model in chapter 10 of Kikuchi and Oden (1988) for Coulomb's law of static friction for the slip case. For a flat contact surface with normal (compressive) and tangential stress fields given by σ_*n*_ and **σ**_*T*_ respectively, Coulomb's law of static friction can be expressed by the following relationship between these surface stresses and the tangential displacement field ***u***_*T*_:

$$\begin{array}{l} |\sigma_{T}|<\nu_{F}|\sigma_{n}|\Rightarrow u_{T}=0,\\ |\sigma_{T}|=\nu_{F}|\sigma_{n}|\Rightarrow u_{T}=-\lambda \sigma_{T}\text{ for some }\lambda ⩾0. \end{array}$$

Here, ν_*F*_ is the coefficient of friction in respect to Coulomb's law of friction. Kikuchi and Oden point out that it is not possible to mathematically analyse a variational problem for Coulomb's law using conventional mathematical methods. As a result, the question of existence of solutions to such friction problems remains an open one. To circumvent this difficulty, Kikuchi and Oden propose the following regularized version of the static friction law involving a small parameter ε:

(6) $$-\sigma_{T}=\nu_{F}|\sigma_{n}|\frac{u_{T}}{\left|u_{T}\right|}\text{ if }|u_{T}|⩾\varepsilon ,$$

(7) $$-\sigma_{T}=\nu_{F}|\sigma_{n}|\frac{u_{T}}{\varepsilon }\text{ if }|u_{T}|<\varepsilon .$$

In the limit ε → 0, Coulomb's original law of static friction is recovered. For the slip case, we assume the first condition holds for large tangential displacements which, on converting this to a membrane in contact with a rigid surface in curvilinear coordinates, yields

(8) $$f_{r}^{\beta }(u)+\nu_{F}{\left(u_{\alpha }u^{\alpha }\right)}^{-\frac{1}{2}}u^{\beta }f_{r}^{3}(u)=0\;.$$

We now rearrange the compatibility condition Equation (5) and use Coulomb's law of friction Equation (8) to find a relationship between the coefficient of friction and the external loadings, given by

(9) $$\begin{array}{l} \nu_{F}\int_{\omega }\frac{\left(F_{\text{​}[\text{II}]\beta \gamma }\tau^{\beta \gamma }(u)+g_{r}^{3}-\varrho F_{\text{​}[\text{II}]\beta \gamma }\partial_{t}u^{\beta }\partial_{t}u^{\gamma }\right)}{{\left(u_{\delta }u^{\delta }\right)}^{\frac{1}{2}}}u^{\alpha }w_{\alpha }\;d\omega \\ \;+\int_{\partial \omega }\tau_{0}^{\alpha }w_{\alpha }\;d(\partial \omega )\\ \;+\int_{\omega }g_{r}^{\alpha }w_{\alpha }-\varrho \left(\partial_{tt}u^{\alpha }+\Gamma_{\text{​}\gamma \delta }^{\alpha }\partial_{t}u^{\gamma }\partial_{t}u^{\delta }\right)w_{\alpha }\;d\omega =0\;,\\ \;\forall \;w\in \{v\in H^{1}(\omega )|\epsilon (v)=0\}\;. \end{array}$$

The residual displacement *u*^β^ and $f_{r}^{j}\left(u\right)$ give us five unknowns and Equations (2), (3), and (8) provide us with five equations. Thus, the system is fully determined with boundary conditions Equation (4). Furthermore, Equation (9) provides us with a stronger system as if the coefficient of friction is unknown then a known traction can close the system and vice versa.

While it is a closed system, it is not straightforward to prove that a solution exists for this model. The problem of proving the existence of solutions arises from the function $f_{r}^{\beta }\left(u\right)$, as it is a function of both ***u*** and **∇*****u***. If $f_{r}^{\beta }\left(u\right)$ was purely a function of ***u***, then the existence of solutions may be proved by variational methods for semi-linear elliptic equations (see Badiale and Serra, 2010) but with the **∇***u* dependence, our model is not even a variational problem. Nevertheless, a numerical finite-difference solution is pursued in the next section.

### 2.3. Numerical analysis

To conduct numerical experiments, assume that we are dealing with a surface of revolution case where both the contact surface and the unstressed membrane are parameterized by the same immersion. Let this immersion be $\sigma (x^{1},x^{2})={\left(x^{1},\varphi \left(x^{1}\right)\text{ sin}\left(x^{2}\right),\varphi \left(x^{1}\right)\text{ cos}\left(x^{2}\right)\right)}_{E}$, where *x*^1^ ∈ (0, *l*) and $x^{2}\in \left(-\frac{1}{2}\pi ,0\right)$. To permit changes in lateral curvature we assert that $\varphi \left(x^{1}\right)=r_{0}-16c{\left(l^{-1}x^{1}-\frac{1}{2}\right)}^{4}$, where, initially, to keep the contact area as a surface of positive mean curvature, *c* is a positive parameter with *c* < *r*_0_. Note that *l, r*_0_ are some positive real constants that are specified later. With some calculations, we find the first fundamental form tensor to be $F_{\text{[I]}}=\text{diag}\left({\left(\psi_{1}\right)}^{2},{\left(\psi_{2}\right)}^{2}\right)$, where $\psi_{1}={\left(1+{\left(\varphi \prime \left(x^{1}\right)\right)}^{2}\right)}^{\frac{1}{2}}$ and $\psi_{2}=\varphi \left(x^{1}\right)$. With a few more calculations one can find that

$$\begin{array}{l} \Gamma_{\text{​}11}^{1}={\left(\psi_{1}\right)}^{-1}\partial_{1}\psi_{1}\;,\;F_{\text{​}[\text{II}]1}^{\;\;1}={\left(\psi_{1}\right)}^{-1}\varphi^{\prime \prime }(x^{1}){\left(1+{\left(\varphi^{\prime }(x^{1})\right)}^{2}\right)}^{-1}\;,\\ \Gamma_{\text{​}21}^{2}={\left(\psi_{2}\right)}^{-1}\partial_{1}\psi_{2}\;,\;F_{\text{​}[\text{II}]2}^{\;\;2}=-{\left(\psi_{2}\right)}^{-1}{\left(1+{\left(\varphi^{\prime }(x^{1})\right)}^{2}\right)}^{-\frac{1}{2}}\;, \end{array}$$

where $\Gamma_{\text{​}\alpha \beta }^{\gamma }$ are the Christoffel symbols of the second kind and ***F***_[II]_ is the second fundamental form tensor. Now given that ***u*** = (*u^1^*(*x*^1^, *x*^2^), *u*^2^(*x*^1^, *x*^2^)) is the displacement field, one can derive the following:

$$\begin{array}{l} \nabla_{\text{​}1}u^{1}=\partial_{1}u^{1}+\Gamma_{\text{​}11}^{1}u^{1},\\ \nabla_{\text{​}1}u^{2}=\partial_{1}u^{2}+\Gamma_{\text{​}21}^{2}u^{2},\\ \nabla_{\text{​}2}u^{1}=\partial_{2}u^{1}-{\left(\psi_{1}\right)}^{-2}{\left(\psi_{2}\right)}^{2}\Gamma_{\text{​}21}^{2}u^{2},\\ \nabla_{\text{​}2}u^{2}=\partial_{2}u^{2}+\Gamma_{\text{​}22}^{2}u^{2}\;. \end{array}$$

Now assume that our membrane is subjected to the acceleration of gravity i.e., subject to (0, 0, −*g*)_*E*_ in Cartesian coordinates. With coordinate transforms, from Euclidean to curvilinear, one may re-express acceleration due to gravity in the curvilinear coordinates as *g**J***, where

$$J=\left(-\varphi^{\prime }(x^{1}){\left(\psi_{1}\right)}^{-2}\cos(x^{2}),\varphi^{-1}(x^{1})\sin(x^{2}),-{\left(\psi_{1}\right)}^{-1}\cos(x^{2})\right)\;,$$

and *x*^2^ is the angle that the vector (ψ_1_, ψ_2_, 0) makes with the vector (0, 0, 1)_*E*_.

Now given that ϱ is the mass density, (0, 0, 0) is the acceleration field and $\left(0,{\left(\psi_{2}\right)}^{-1}V,0\right)$ is the velocity field of the membrane, then we can express the governing equations of the membrane as

$$\begin{array}{l} \;(\Lambda +\mu )\partial^{1}\left(\nabla_{\text{​}\alpha }u^{\alpha }\right)+\mu \Delta u^{1}+\varrho gJ^{1}+f_{r}^{1}(u)=-\varrho {\left(\psi_{1}\right)}^{-2}\Gamma_{\text{​}21}^{2}V^{2}\;,\\ (\Lambda +\mu )\partial^{2}\left(\nabla_{\text{​}\alpha }u^{\alpha }\right)+\mu \Delta u^{2}+\varrho gJ^{2}+f_{r}^{2}(u)=0\;, \end{array}$$

and

$$\begin{array}{l} \left((\Lambda +2\mu )\left(\partial_{1}u^{1}+\Gamma_{\text{​}11}^{1}u^{1}\right)+\Lambda \left(\partial_{2}u^{2}+\Gamma_{\text{​}21}^{2}u^{1}\right)\right)F_{\text{​}[\text{II}]1}^{\;\;1}\\ \;\;+\left(\Lambda \left(\partial_{1}u^{1}+\Gamma_{\text{​}11}^{1}u^{1}\right)+(\Lambda +2\mu )\left(\partial_{2}u^{2}+\Gamma_{\text{​}21}^{2}u^{1}\right)\right)\\ \;F_{\text{​}[\text{II}]2}^{\;\;2}+\varrho gJ^{3}\;+\;f_{r}^{3}(u)=\varrho F_{\text{​}[\text{II}]2}^{\;\;2}V^{2}\;, \end{array}$$

where Λ = 2λμ(λ + 2μ)^−1^ and λ and μ are the first and second Lamé parameters respectively. Assuming that our contact area is rough, the final governing equation required is the friction law Equation (8) where the coefficient of friction ν_*F*_ is considered to be an unknown.

Now divide the boundary into sub-boundaries, so that

$$\begin{array}{l} \;\partial \omega_{f}=\left\{\{0\}\times \left(-\frac{1}{2}\pi ,0\right)\right\}\cup \left\{\{l\}\times \left(-\frac{1}{2}\pi ,0\right)\right\}\;\\ \;\partial \omega_{T_{0}}=\left\{[0,l]\times \left\{-\frac{1}{2}\pi \right\}\right\}\;,\\ \partial \omega_{T_{\text{max}}}=\{\{[0,l]\times \{0\}\}\;, \end{array}$$

and we assert that the boundary conditions are

$$\begin{array}{l} {\left(\psi_{1}\right)}^{2}\partial_{2}u^{1}+{\left(\psi_{2}\right)}^{2}\partial_{1}u^{2}{|}_{\partial \omega }=0\;(\text{zero}-\text{Robin})\;,\\ (\Lambda +2\mu )\left(\partial_{1}u^{1}+\Gamma_{\text{​}11}^{1}u^{1}\right)+\Lambda \left(\partial_{2}u^{2}+\Gamma_{\text{​}21}^{2}u^{1}\right){|}_{\partial \omega_{f}}\\ \;=0\;(\text{zero}-\text{Robin})\;,\\ \Lambda \left(\partial_{1}u^{1}+\Gamma_{\text{​}11}^{1}u^{1}\right)+(\Lambda +2\mu )\left(\partial_{2}u^{2}+\Gamma_{\text{​}21}^{2}u^{1}\right){|}_{\partial \omega_{T_{0}}}\\ \;=\tau_{0}\;(\text{traction})\;,\\ \Lambda \left(\partial_{1}u^{1}+\Gamma_{\text{​}11}^{1}u^{1}\right)+(\Lambda +2\mu )\left(\partial_{2}u^{2}+\Gamma_{\text{​}21}^{2}u^{1}\right){|}_{\partial \omega_{T_{\text{max}}}}\\ \;=\tau_{\text{max}}\;(\text{traction})\;, \end{array}$$

where τ_max_ > τ_0_ are positive real constants.

Finally, we take Equation (9) and modify it ever so slightly to make it easier to numerically model, thus obtaining the following relation,

(10) $$\begin{array}{l} \left(\tau_{\text{max}}-\tau_{0}\right)\int_{0}^{l}\psi_{1}\psi_{2}\;dx^{1}\\ \;+\varrho \int_{\omega }\left(gJ^{\alpha }\psi_{\alpha }+{\left(\psi_{1}\right)}^{-1}\Gamma_{\text{​}21}^{2}V^{2}\right)\psi_{1}\psi_{2}\;dx^{1}dx^{2}\\ \;-\sqrt{2}\;\nu_{F}\text{​​}\int_{\omega }f_{r}^{3}(u)\psi_{1}\psi_{2}\;dx^{1}dx^{2}=0\;. \end{array}$$

Now we are ready to conduct some numerical experiments. Our goal in this section is to investigate how variables such as the Gaussian curvature, Young's modulus, Poisson's ratio, speed and the mass density of the membrane and the traction may affect the value of the coefficient of friction obtained. Note that for our experiments, we keep the values τ_0_ = 1, *l* = 1, *r*_0_ = 1 and *g* = 9.81 fixed.

To conduct numerical experiments, we employ a second-order accurate finite-difference method in conjunction with Newton's method for nonlinear systems. On discretising the domain, as we are dealing with curvilinear coordinates, we find that $\Delta x^{2}\leq \psi_{0}\Delta x^{1}$, for all $\psi_{0}\in \left\{\psi_{1}/\psi_{2}|x^{1}\in \left[0,l\right]\right\}$ where Δ*x*^β^ is a small increment in the *x*^β^ direction. For our purposes we use $\Delta x^{2}=\frac{1}{N-1}$ and $\psi_{0}=\psi_{1}/\psi_{2}{|}_{x^{1}=\frac{1}{2}l}$, where *N* = 250. We also choose to terminate our iterating process once |1 − ν_*Fm*+1_/ν_*Fm*_| falls below a certain specified tolerance, where ν_*Fm*_ is the *m*^th^ iterative solution for the coefficient of friction. Note that to numerically model Equation (10), we use the prismoidal formula (Meserve and Pingry, 1952). Unfortunately, as this is a pure-traction problem iterative schemes can be highly unstable and so, to ensure convergence, the condition $u^{2}{|}_{\partial \omega_{T_{0}}}\leq 0$ is strictly enforced.

We initially ran the numerical code using the following values: $\tau_{\text{max}}=\frac{3}{2}$, *c* = 0, *E* = 10^3^, $\nu =\frac{1}{4}$, *V* = 0.01, ϱ = 0.01 and with a grid of 160 × 250 points. The grid size *N* was varied to confirm accuracy of the converged solution. The coefficient of friction calculated in this case is ν_*F*_ = 0.195 to three significant figures. We now proceed to investigate how the variation in certain parameters may change this value and thus see whether this differs from the value predicted by the classical capstan model Equation (1).

**Figure 4:**
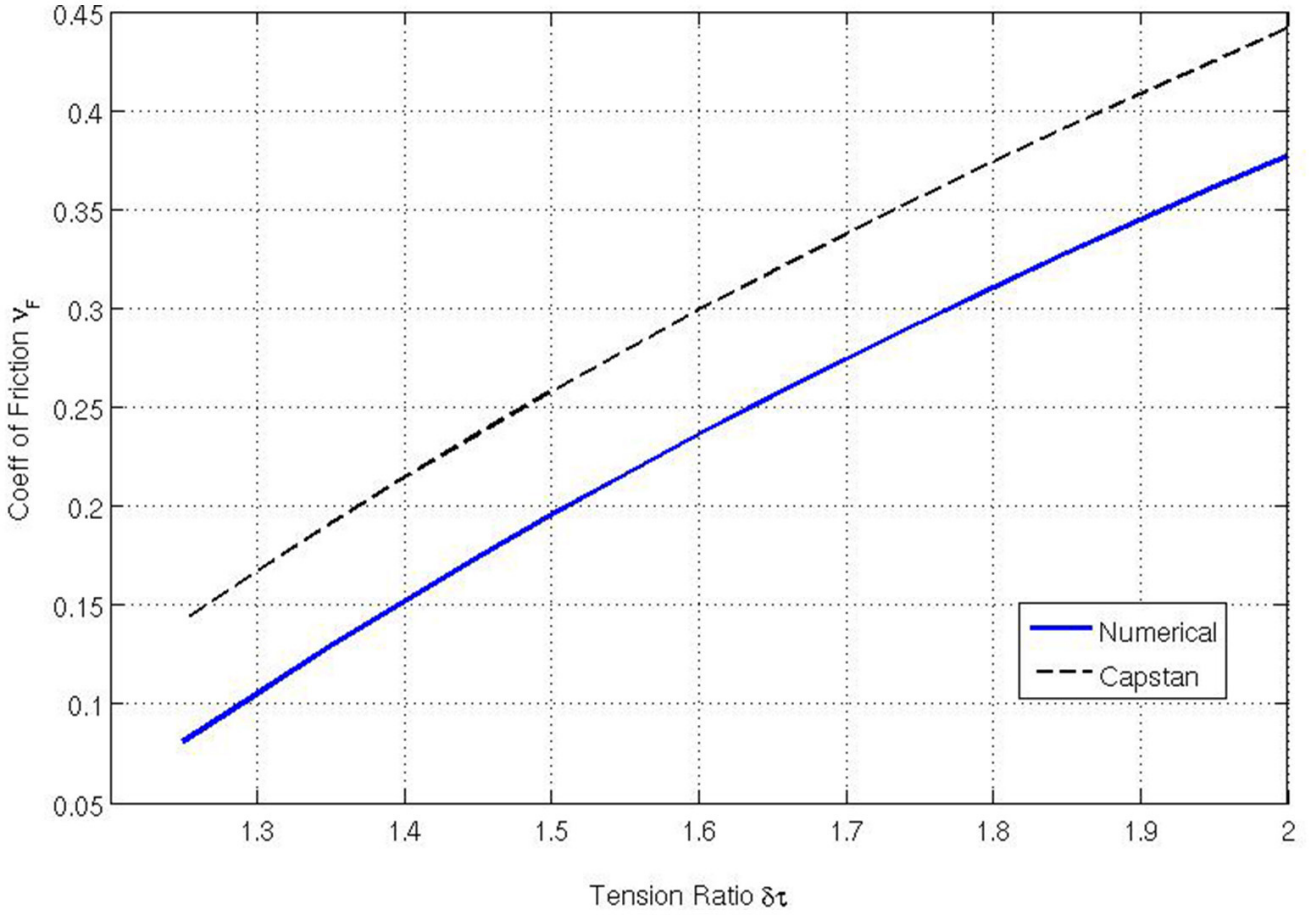
**Coefficient of friction relative to δτ = τ_**max**_/τ_**0**_ calculated using our numerical model and compared to the standard capstan result: $\nu_{F}=\frac{2}{\pi }\;\ln\;\delta \tau$**.

We begin by varying the tension applied to the membrane. Figure 4 is calculated with the values of τ_max_ ∈ {1.25, 1.30, 1.35, … 2.00}, *c* = 0, *E* = 10^3^, $\nu =\frac{1}{4}$, *V* = 0.01 and ϱ = 0.01. It shows that as the tension ratio increases, the coefficient of friction also increases. This is intuitive because, as the maximum applied tension increases, the coefficient of friction must increase to maintain a constant speed. Equation (1) predicts a similar trend and so agreement with the classical capstan model seems quite good although the figure shows that the numerical code produces consistently lower values. The principal reason for the discrepancy is the non-zero mass density of the fabric which is investigated below.

**Figure 5:**
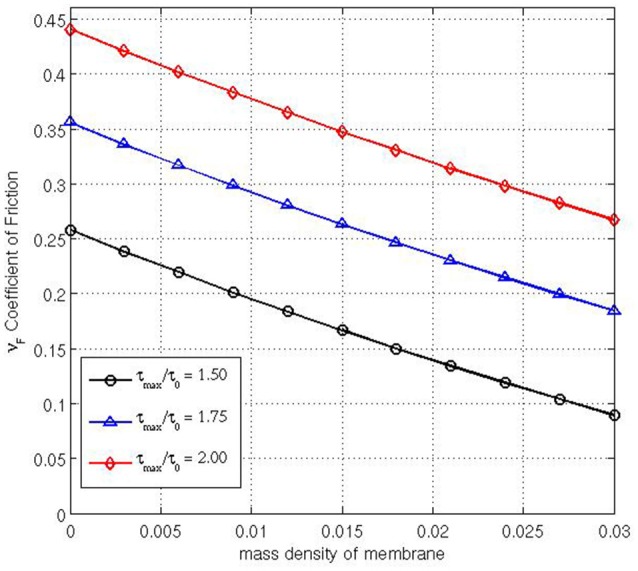
**Coefficient of friction relative to ρ for three different values of τ_**max**_/τ_**0**_ = [1.50,1.75,2.00]**.

Varying Poisson's ratio ν, the Young's modulus *E* and the speed of the membrane *V* do not lead to any significant changes in the coefficient of friction. But when we examine varying the mass density of the fabric we find significant alterations to the coefficient of friction determined by the model. Figure 5 is calculated for three different applied tensions: τ_max_ ∈ {1.50, 1.75, 2.00}, with values *c* = 0, *E* = 10^3^, $\nu =\frac{1}{4}$, *V* = 0.01 and ϱ ∈ {0, 0.003, 0.006, …, 0.03}. This shows as the mass density (with respect to the volume) of the membrane increases, the coefficient of friction decreases markedly in all cases. As ρ → 0 the coefficient of friction obtained from Equation (1), e.g., (2/π) ln(3/2) = 0.258… , is attained, but even a small mass density, ρ = O(10^−3^) for instance, can lead to the accuracy being significantly reduced to only one significant figure. This feature is ignored in typical capstan equation calculations.

**Figure 6:**
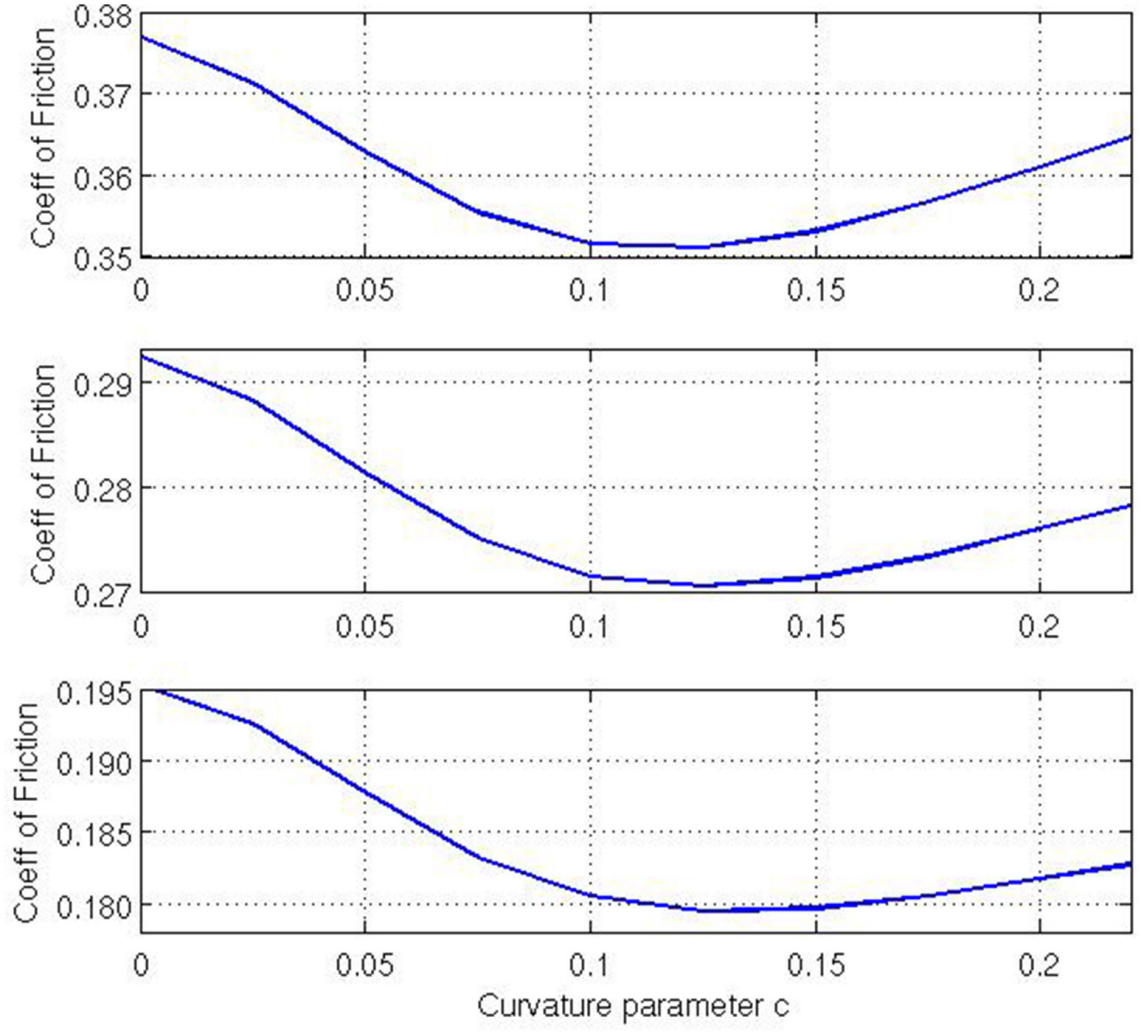
**Coefficient of friction relative to lateral curvature parameter ***c*** where the geometry has non-negative Gaussian curvative. Top**: τ_max_/τ_0_ = 2.00, **Middle**: τ_max_/τ_0_ = 1.75, **Bottom**: τ_max_/τ_0_ = 1.50.

**Figure 7:**
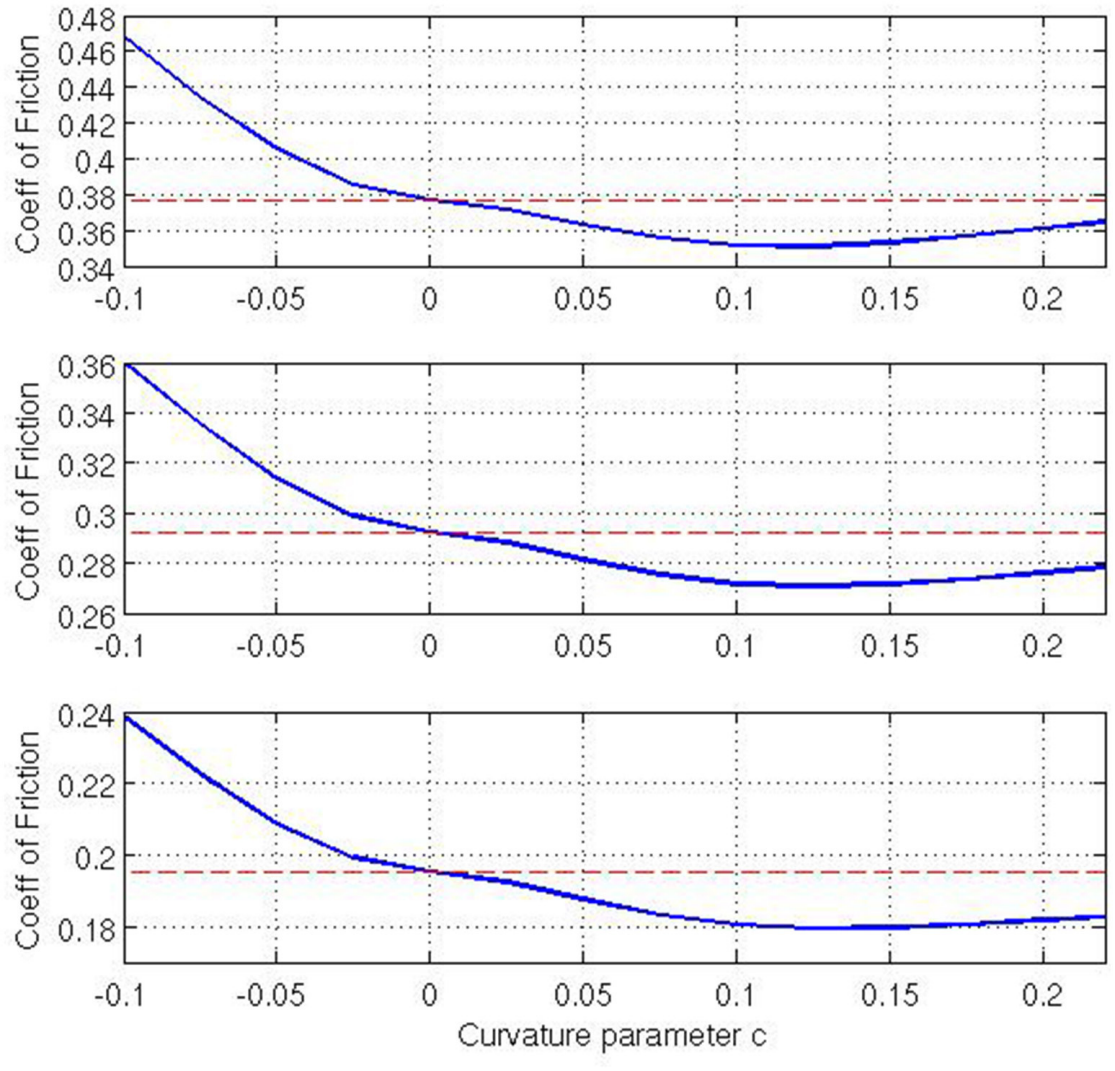
**Coefficient of friction relative to ***c*** including geometries with positive and negative Gaussian curvature. Top:** τ_max_/τ_0_ = 2.00, **Middle**: τ_max_/τ_0_ = 1.75, **Bottom**: τ_max_/τ_0_ = 1.50.

Finally, let us examine what effect varying the Gaussian curvature *K* has on the coefficient of friction. Note that, for our given geometry, *K* is a function of *x*^1^, and thus it varies across the contact surface. As our curvature parameter *c* changes the Gaussian curvature changes across the contact surface. For *c* = 0, *K* = 0 everywhere. For *c* > 0 the contact surface is a barrel-shape with non-negative (i.e., positive or zero) Gaussian curvature whereas for *c* < 0 the contact surface is a saddle-shape with non-positive (i.e., negative or zero) Gaussian curvature across the contact area. This implies that there exists a positive correlation between *c* and *K*, i.e., as *c* increases so does *K* and so we investigate how *c* relates to ν_*F*_. Figure 6 is calculated for non-negative Gaussian curvatures with the values of τ_max_ ∈ {1.50, 1.75, 2.00}, and $c\in \left\{0,\frac{1}{40},\frac{2}{40},\frac{3}{40},\frac{4}{40},\frac{5}{40},\frac{6}{40},\frac{7}{40},\frac{8}{40},\frac{9}{40}\right\}$, *E* = 10^3^, $\nu =\frac{1}{4}$, *V* = 0.01, and ϱ = 0.01. The three plots in the figure for different tension ratios demonstrate that as *c* increases (i.e., Gaussian curvature increases) the coefficient of friction decreases and there exists an optimum value of *c*, where the coefficient of friction is at a minimum, given that all other variables are constant. For our simulations, this value is observed around $c=\frac{1}{8}$. The initial decrease in ν_*F*_ as *c* increases is intuitive. To illustrate this, consider a membrane pulled over a surface with high curvature. The higher curvature would leads to higher normal reaction force which, in turn, results in a higher friction force and finally a higher maximum applied tension. Also inclusion of a nonzero lateral curvature (note that we are considering a case with two positive principal curvatures) means that a higher maximum applied tension is required to support the strains in the lateral direction. Thus, for even a relatively small coefficient of friction, a higher maximum applied tension can be observed. Hence, if one kept every variable fixed, except for the Gaussian curvature and the coefficient of friction, then one would expect to see a low coefficient of friction for a high Gaussian curvature. However, the existence of a minimum ν_*F*_ for positive *c* is a surprising outcome suggesting a possible optimum Gaussian curvature that can lead to a minimum coefficient of friction. Conversely, it is possible numerically to extend our range of *c* values to include $\left\{-\frac{1}{40},-\frac{2}{40},-\frac{3}{40},-\frac{4}{40}\right\}$ that represent saddle-type contact regions. These simulations suggest that such saddle-type contact regions lead to significantly higher values of ν_*F*_ than for the zero-curvature case (Figure 7). Extending that range yet further to *c* = −0.02 (not shown) suggests that no *maximum* coefficient of friction is attained but that the coefficient of friction continues to increase as *c* becomes more negative. Such intriguing variations in ν_*F*_ captured by our model here cannot be simulated by the classical capstan model Equation (1).

## 3. Shell-membranes supported by elastic foundations with static friction

In this section, we examine the effect that the deformation of the underlying substrate has on the frictional forces generated in the region of contact. To do this, we derive a shell-membrane model to describe the behavior of an overlying compliant sheet on an elastic foundation subjected to static friction. However, such a computation requires our frictional law (e.g., Coulomb's law of static friction) to be imposed on a *free boundary* as the displacement across the contact region is unknown.

### 3.1. Deriving a displacement-based frictional law

Recall Kikuchi and Oden's model (chapter 10 of Kikuchi and Oden, 1988) for Coulomb's law of friction for slip that we extended to curvilinear coordinates Equation (8). For the static case, by eliminating the regularization parameter ε from their original Equations, (6) and (7), the friction law can be expressed in the following form

$$T_{3}^{\beta }(u)+\nu_{F}{\left(g_{33}\right)}^{\frac{1}{2}}{\left(u_{\alpha }u^{\alpha }\right)}^{-\frac{1}{2}}T_{3}^{3}(u)u^{\beta }\leq 0.$$

Unlike the rigid substrate case, this equation is extremely difficult to impose on a free contact boundary between shell-membrane and substrate in its present form. Therefore, a more computationally tractable displacement-based approximation is sought. Assume that *u*^β^ ≥ 0 and contract the above equation with *u*^β^. Noting that in our framework *g*_33_ = 1, we thus find

$$\begin{array}{l} \mu \left(u^{\alpha }{\bar{\nabla }}_{\text{​}\alpha }u^{3}+\frac{1}{2}{\bar{\nabla }}_{\text{​}3}(u_{\alpha }u^{\alpha })\right)+\nu_{F}{\left(u_{\alpha }u^{\alpha }\right)}^{\frac{1}{2}}\\ \;\left(\lambda {\bar{\nabla }}_{\text{​}\alpha }u^{\alpha }+(\lambda +2\mu ){\bar{\nabla }}_{\text{​}3}u^{3}\right)\leq 0\;. \end{array}$$

Now assume that this body is in contact with an elastic foundation, thus it permits normal displacements at the boundary, so we assert that only normal derivatives are of any consequence. Hence, we may approximate the above relation as

$$\mu {\bar{\nabla }}_{\text{​}3}{\left(u_{\alpha }u^{\alpha }\right)}^{\frac{1}{2}}+\nu_{F}(\lambda +2\mu ){\bar{\nabla }}_{\text{​}3}u^{3}\leq 0\;.$$

To simplify matters further, we assert that the condition is independent of any elastic properties of the overlying body. This may be achieved by assuming λ = 0, and thus we find

$${\bar{\nabla }}_{\text{​}3}\left({\left(u_{\alpha }u^{\alpha }\right)}^{\frac{1}{2}}+2\nu_{F}u^{3}\right)\leq 0\;.$$

By approximating the above condition even further, we arrive at the following hypothesis:

Hypothesis 1. *A shell supported by an elastic foundation with a rough contact area, that has thickness small relative to the radius of mean curvature, satisfies the following displacement-based friction condition*

(11) $$u^{3}\leq -\frac{1}{2\nu_{F}}{\left(u_{\alpha }u^{\alpha }\right)}^{\frac{1}{2}}\;,$$

*where* ν_*F*_ *is the coefficient of friction between shell and the foundation, and **u** is the displacement field of the shell. If* $\;2\nu_{F}u^{3}+{\left(u_{\alpha }u^{\alpha }\right)}^{\frac{1}{2}}<0$, *then we say that the the shell is bonded to the foundation, and if* $\;2\nu_{F}u^{3}+{\left(u_{\alpha }u^{\alpha }\right)}^{\frac{1}{2}}=0$, *then we say that the shell is at limiting equilibrium*.

The justifications for introducing the hypothesis are as follows. Kikuchi and Oden (1988) assert that the variational form of a linear elastic body subjected to Coulomb's law of static friction, is non-convex and non-differentiable. Thus, the existence of a (unique or otherwise) solution is an open question that cannot be proven with conventional means. But we can show that the variational form, i.e., the energy functional of a linear elastic body, subjected to the displacement-based friction condition from this hypothesis is convex, coercive and differentiable, and thus proving the existence of solutions is perfectly possible. Also, unlike the model of Kikuchi and Oden (1988), our displacement-based condition is independent of the regularization parameter ε and it is well defined for all finite values of ***u***. Furthermore, we show that our problem can be numerically modeled without an initial guess of the purely normal stress, which is something Kikuchi and Oden's model is incapable of. Note, however, that we can guarantee that the condition from our hypothesis will hold as we have already asserted that the lower-surface of the shell is not hyperbolic and its mean curvature is positive. Thus, for sensible boundary conditions, we can always expect the normal displacement to be non-positive.

Does our hypothesized displacement-based friction law make physical sense? Well, consider two elastic blocks in a zero-gravity scenario, block-*A* at the top and block-*B* at the bottom, where the base of block-*B* satisfies the zero-Dirichlet boundary condition. Now assume that the contact area of both blocks is rough and that one is applying forces to both the top and to one side of block-*A* to mimic respectively compression and shear in the contact region. The higher the compression, the higher the normal displacement is toward the bottom, i.e., *u*^3^ < 0, and, the higher the shear, the higher the tangential displacement is in the direction of the applied tangential force. Just as for the case of Coulomb's friction, where the bodies are in respective equilibrium given that the magnitude of the normal stress is above a certain factor of the magnitude of the tangential stress, i.e., $\left|T_{3}^{3}\left(u\right)\right|>\nu_{F}^{-1}{\left|T_{3}^{\alpha }\left(u\right)T_{\alpha }^{3}\left(u\right)\right|}^{\frac{1}{2}}$, we assert that the bodies are in equilibrium given that the normal displacement is below a certain factor of the magnitude of the tangential displacement, i.e., $u^{3}<-\frac{1}{2}\nu_{F}^{-1}{\left(u_{\alpha }u^{\alpha }\right)}^{\frac{1}{2}},\text{ if }u^{3}<0$. Note that this factor may or may not be $\frac{1}{2}\nu_{F}^{-1}$, but this is the most mathematically logical factor we have derived.

### 3.2. The governing equations

Let Ω ⊂ ℝ^3^ be a connected open bounded domain that satisfies the segment condition with a uniform *C*^1^(ℝ^3^; ℝ^2^) boundary ∂Ω such that ω, ∂Ω_0_ ⊂ ∂Ω with $\bar{\omega }\;\cap \;\bar{\partial \;\Omega_{0}}=$ Ø and meas$\left(\partial \Omega_{0};{\mathbb{R}}^{2}\right)>0$, and let ω ⊂ ℝ^2^ be a connected open bounded plane that satisfies the segment condition with a uniform *C*^1^(ℝ^2^; ℝ) boundary ∂ω. Let $\bar{X}\in C^{2}\left(\bar{\Omega };{\text{E}}^{3}\right)$ be a diffeomorphism and $\sigma \in C^{3}\left(\bar{\omega };{\text{E}}^{3}\right)$ be an injective immersion. Let ***f*** ∈ ***L***^2^(Ω), $f_{0}\in L^{2}\left(\omega \right)$ and $\tau_{0}\in L^{2}\left(\partial \omega \right)$.

For this section, we assume that ***u*** ∈ *C*^2^(Ω; ℝ^3^), $u^{\alpha }{|}_{\omega }\in C^{3}\left(\omega \right)$, $u^{3}{|}_{\omega }\in C^{4}\left(\omega \right)$ and $2\nu_{F}u^{3}+{\left(u_{\alpha }u^{\alpha }\right)}^{\frac{1}{2}}\leq 0$ everywhere in ω. For the elastic foundation we define $T^{ij}\left(u\right)=A^{ijkl}E_{kl}\left(u\right)$ to be the second Piola-Kirchhoff stress tensor, $E_{ij}\left(u\right)=\frac{1}{2}\left(g_{ik}{\bar{\nabla }}_{j}u^{k}+g_{jk}{\bar{\nabla }}_{i}u^{k}\right)$ to be the linearized Green-St Venant stress tensor, $A^{ijkl}=\bar{\lambda }g^{ij}g^{kl}+\bar{\mu }\left(g^{ik}g^{jl}+g^{il}g^{jk}\right)$ to be the elasticity tensor, $\bar{\lambda }={\left(1-\bar{\nu }-2{\bar{\nu }}^{2}\right)}^{-1}\bar{\nu }Ē$ as the first Lamé parameter, $\bar{\mu }=\frac{1}{2}{\left(1+\bar{\nu }\right)}^{-1}Ē$ as the second Lamé parameter, *Ē* as the Young's modulus and $\bar{\nu }$ as Poisson's ratio of the elastic foundation. Furthermore, ***f*** is some external force density field acting on the elastic foundation. The covariant *first fundamental form tensor* of **σ** with respect to the curvilinear coordinates is defined as

$$F_{\text{​}[\text{I}]\alpha \beta }=\partial_{\alpha }\sigma_{i}\partial_{\beta }\sigma^{i}\;,\;\forall \;\alpha ,\beta \in \{1,2\}\;.$$

Also we regard the indices α, β, γ, δ ∈ {1, 2}. Furthermore, $F_{\text{[I]}\alpha \gamma }F_{\text{[I]}}^{\gamma \beta }=\delta_{\alpha }^{\beta }$, ∀ α, β ∈ {1, 2}. The second fundamental form tensor is as defined above in Section 2.2.

The governing equations of the elastic foundation are given by

$${\bar{\nabla }}_{\text{​}i}T_{j}^{i}(u)+f_{j}=0\;,\;\forall \;j\in \{1,2,3\}\;,$$

with the following boundary conditions:

$$\begin{array}{l} \;u{|}_{\partial \Omega_{0}}=0\;,\\ {\bar{n}}_{i}T_{j}^{i}(u){|}_{\left\{\partial \Omega \setminus \{\omega \cup \partial \Omega_{0}\}\right\}}=0\;,\;\forall \;j\in \{1,2,3\}\;, \end{array}$$

where $\bar{n}$ is the unit outward normal to the boundary ∂Ω in curvilinear coordinates.

Turning now to the overlying body, we assume that the body is so thin, and the bending moments are so small, that it can be approximated by a shell-membrane. For the shell-membrane we define $\tau^{\alpha \beta }\left(u\right)=B^{\alpha \beta \gamma \delta }\epsilon_{\gamma \delta }\left(u\right)$ to be the stress tensor and $\eta^{\alpha \beta }\left(u\right)=B^{\alpha \beta \gamma \delta }\rho_{\gamma \delta }\left(u\right)$ to be the negative of the change in the moments density tensor.

$$\epsilon_{\alpha \beta }(u)=\frac{1}{2}(\nabla_{\text{​}\alpha }(u_{\beta }{|}_{\omega })+\nabla_{\text{​}\beta }(u_{\alpha }{|}_{\omega }))-F_{\text{​}[\text{II}]\alpha \beta }(u^{3}{|}_{\omega })$$

is then half the change in the first fundamental form tensor,

$$\begin{array}{l} \rho_{\alpha \beta }(u)=\nabla_{\text{​}\alpha }\nabla_{\text{​}\beta }(u^{3}{|}_{\omega })-F_{\text{​}[\text{II}]\alpha \gamma }F_{\text{​}[\text{II}]\beta }^{\;\;\gamma }(u^{3}{|}_{\omega })+F_{\text{​}[\text{II}]\beta \gamma }\nabla_{\text{​}\alpha }(u^{\gamma }{|}_{\omega })\\ \;+\;F_{\text{​}[\text{II}]\alpha \gamma }\nabla_{\text{​}\beta }(u^{\gamma }{|}_{\omega })+\;\left(\nabla_{\text{​}\alpha }F_{\text{​}[\text{II}]\beta \gamma }\right)(u^{\gamma }{|}_{\omega }) \end{array}$$

is the change in the second fundamental form tensor,

$$B^{\alpha \beta \gamma \delta }=\frac{2\lambda \mu }{\lambda +2\mu }F_{\text{​}[\text{I}]}^{\alpha \beta }F_{\text{​}[\text{I}]}^{\gamma \delta }+\mu (F_{\text{​}[\text{I}]}^{\alpha \gamma }F_{\text{​}[\text{I}]}^{\beta \delta }+F_{\text{​}[\text{I}]}^{\alpha \delta }F_{\text{​}[\text{I}]}^{\beta \gamma })$$

is the elasticity tensor, λ = (1 − ν − 2ν^2^)^−1^ ν*E* is the first Lamé parameter, $\mu =\frac{1}{2}{\left(1+\nu \right)}^{-1}E$ is the second Lamé parameter, *E* is the Young's modulus and ν is Poisson's ratio of the frictionally coupled shell. Tr$\left(T_{j}^{3}\left(u\right)\right)=T_{j}^{3}\left(u\right){|}_{\omega }$ is the normal stress of the foundation in the contact region, and ***f***_0_ is some external force density field acting on the overlying shell.

The governing equations for the shell-membrane are determined by considering a variational problem based on the following energy functional:

$$\begin{array}{l} J(u)=\int_{\Omega }\frac{1}{2}A^{ijkl}E_{ij}(u)E_{kl}(u)-f^{i}u_{i}\;d\Omega \\ \;+\int_{\omega }\frac{1}{2}B^{\alpha \beta \gamma \delta }\left(h\epsilon_{\alpha \beta }(u)\epsilon_{\gamma \delta }(u)+\frac{1}{3}h^{3}\rho_{\alpha \beta }(u)\rho_{\gamma \delta }(u)\right)\\ \;-hf_{0}^{i}u_{i}\;d\omega -\int_{\partial \omega }h\tau_{\text{​}0}^{i}u_{i}\;d(\partial \omega )\;, \end{array}$$

where ***u*** is subject to the displacement-based friction condition Equation (11) and the region in which the shell is at limiting equilibrium is unknown prior to solving the problem. Following the detailed analysis in chapter 4 of Jayawardana (2016), it is possible to obtain the following governing equations for the shell-membrane:

**if** $[2\nu_{F}u^{3}+{\left(u_{\alpha }u^{\alpha }\right)}^{\frac{1}{2}}]{|}_{\omega }<0$ (the *bonded* case), **then**

$$\begin{array}{l} \nabla_{\text{​}\alpha }\tau_{\beta }^{\alpha }(u)+\frac{2}{3}h^{2}F_{\text{​}[\text{II}]\beta }^{\;\;\alpha }\nabla_{\text{​}\gamma }\eta_{\alpha }^{\gamma }(u)+\frac{1}{3}h^{2}\left(\nabla_{\text{​}\gamma }F_{\text{​}[\text{II}]\beta }^{\;\;\alpha }\right)\eta_{\alpha }^{\gamma }(u)\\ \;-\frac{1}{h}\text{Tr}(T_{\beta }^{3}(u))+f_{0\beta }=0\;,\;\forall \;\beta \in \{1,2\}\;,\\ F_{\text{​}[\text{II}]\alpha }^{\;\;\gamma }\tau_{\gamma }^{\alpha }(u)-\frac{1}{3}h^{2}\nabla_{\text{​}\alpha }\left(\nabla_{\text{​}\gamma }\eta^{\alpha \gamma }(u)\right)+\frac{1}{3}h^{2}F_{\text{​}[\text{II}]\alpha }^{\;\;\delta }F_{\text{​}[\text{II}]\gamma }^{\;\;\alpha }\eta_{\delta }^{\gamma }(u)\\ \;-\frac{1}{h}\text{Tr}(T_{3}^{3}(u))+f_{03}=0\;; \end{array}$$

**if** $[2\nu_{F}u^{3}+{\left(u_{\alpha }u^{\alpha }\right)}^{\frac{1}{2}}]{|}_{\omega }=0$ (the *limiting equilibrium* case), **then**

$$\begin{array}{l} \nu_{F}\nabla_{\text{​}\alpha }\tau_{\beta }^{\alpha }(\bar{u})-\frac{1}{2}\frac{u_{\beta }\;}{{\left(u_{\alpha }u^{\alpha }\right)}^{\frac{1}{2}}}F_{\text{​}[\text{II}]\alpha }^{\;\;\gamma }\tau_{\gamma }^{\alpha }(\bar{u})\\ \;+\frac{2}{3}\nu_{F}h^{2}F_{\text{​}[\text{II}]\beta }^{\;\;\alpha }\nabla_{\text{​}\gamma }\eta_{\alpha }^{\gamma }(\bar{u})+\frac{1}{6}h^{2}\frac{u_{\beta }\;}{{\left(u_{\alpha }u^{\alpha }\right)}^{\frac{1}{2}}}\nabla_{\text{​}\alpha }\nabla_{\text{​}\gamma }\eta^{\alpha \gamma }(\bar{u})\\ \;+\frac{1}{3}\nu_{F}h^{2}\left(\nabla_{\text{​}\gamma }F_{\text{​}[\text{II}]\beta }^{\;\;\alpha }\right)\eta_{\alpha }^{\gamma }(\bar{u})\\ \;-\frac{1}{6}h^{2}\frac{u_{\beta }\;}{{\left(u_{\alpha }u^{\alpha }\right)}^{\frac{1}{2}}}F_{\text{​}[\text{II}]\alpha }^{\;\;\delta }F_{\text{​}[\text{II}]\gamma }^{\;\;\alpha }\eta_{\delta }^{\gamma }(\bar{u})\\ \;-\frac{\nu_{F}}{h}\text{Tr}(T_{\beta }^{3}(\bar{u}))+\frac{1}{2h}\frac{u_{\beta }\;}{{\left(u_{\alpha }u^{\alpha }\right)}^{\frac{1}{2}}}\text{Tr}(T_{3}^{3}(\bar{u}))\\ \;+\nu_{F}f_{0\beta }-\frac{1}{2}\frac{u_{\beta }\;}{{\left(u_{\alpha }u^{\alpha }\right)}^{\frac{1}{2}}}f_{03}=0\;,\;\;\;\;\;\;\;\;\;\;\;\;\;\;\;\;\forall \;\beta \in \{1,2\}\;, \end{array}$$

where $\bar{u}{|}_{\omega }=(u^{1},u^{2},-\frac{1}{2}\nu_{F}^{-1}{\left(u_{\alpha }u^{\alpha }\right)}^{\frac{1}{2}}){|}_{\omega }\text{and }(\partial_{3}{\bar{u}}^{1},\partial_{3}{\bar{u}}^{2},\partial_{3}{\bar{u}}^{3}){|}_{\omega }=(\partial_{3}u^{1},\partial_{3}u^{2},\partial_{3}u^{3}){|}_{\omega }$. Finally, the boundary conditions of the overlying shell are

$$\begin{array}{l} {}[n_{\alpha }\tau_{\beta }^{\alpha }(u)+\frac{2}{3}h^{2}n_{\gamma }F_{\text{​}[\text{II}]\beta }^{\;\;\alpha }\eta_{\alpha }^{\gamma }(u)]{|}_{\partial \omega }=\tau_{\text{​}0\beta }\;,\;\forall \;\beta \in \{1,2\}\;,\\ \;-\frac{1}{3}h^{2}n_{\gamma }\nabla_{\text{​}\alpha }\eta^{\alpha \gamma }(u){|}_{\partial \omega }=\tau_{\text{​}03}\;,\\ \;\partial_{\beta }(u^{3}{|}_{\omega }){|}_{\partial \omega }=0\;,\;\forall \;\beta \in \{1,2\}\;, \end{array}$$

where ***n*** is the unit outward normal vector to the boundary ∂ω in curvilinear coordinates and **τ**_0_ is the external traction field acting on the boundary of the overlying shell.

### 3.3. Numerical experiments

To conduct numerical experiments, assume that we are dealing with a shell-membrane of thickness *h*, supported by an elastic foundation, where the unstrained configuration of the foundation has an annular cross-section, characterized by the diffeomorphism $\bar{X}(x,\theta ,r)={\left(x,r\text{ sin}(\theta ),r\text{ cos}(\theta )\right)}_{\text{E}},\text{where }(x^{1},x^{2},x^{3})=(x,\theta ,r),x\in (-L,L),\theta \in (-\pi ,\pi ],\text{ and }r\in (a_{0},a)$, and assume that the contact region lies within *x* ∈ (−ℓ, ℓ), $\theta \in \left(-\frac{1}{2}\pi ,0\right)$, where 0 < ℓ < *L*. Let the sufficiently smooth field ***u*** = (*u*^1^(*x*, θ, *r*), *u*^2^(*x*, θ, *r*), *u*^3^(*x*, θ, *r*)) be the displacement field of the foundation. With some calculations one can find the metric tensor to be ***g*** = diag(1, *r*^2^, 1) and the covariant derivatives to be

$$\begin{array}{l} {\bar{\nabla }}_{\text{​}1}u^{1}=\partial_{1}u^{1},\;\;{\bar{\nabla }}_{\text{​}1}u^{2}=\partial_{1}u^{2},\;\;{\bar{\nabla }}_{\text{​}1}u^{3}=\partial_{1}u^{3},\\ {\bar{\nabla }}_{\text{​}2}u^{1}=\partial_{2}u^{1},\;\;{\bar{\nabla }}_{\text{​}2}u^{2}=\partial_{2}u^{2}+r^{-1}u^{3},\;\;\;{\bar{\nabla }}_{\text{​}2}u^{3}=\partial_{2}u^{3}-ru^{2},\\ {\bar{\nabla }}_{\text{​}3}u^{1}=\partial_{3}u^{1},\;\;{\bar{\nabla }}_{\text{​}3}u^{2}=\partial_{3}u^{2}+r^{-1}u^{2},\;\;{\bar{\nabla }}_{\text{​}3}u^{3}=\partial_{3}u^{3}. \end{array}$$

With further calculations, one can express the governing equations of the foundation as

$$\begin{array}{l} (\bar{\lambda }+\bar{\mu })\partial^{1}({\bar{\nabla }}_{\text{​}i}u^{i})+\bar{\mu }\bar{\Delta }u^{1}=0\;,\\ (\bar{\lambda }+\bar{\mu })\partial^{2}({\bar{\nabla }}_{\text{​}i}u^{i})+\bar{\mu }\bar{\Delta }u^{2}=0\;,\\ (\bar{\lambda }+\bar{\mu })\partial^{3}({\bar{\nabla }}_{\text{​}i}u^{i})+\bar{\mu }\bar{\Delta }u^{3}=0\;. \end{array}$$

The boundary of the foundation can be decomposed into sub-boundaries as

$$\begin{array}{l} \;\partial \Omega =\bar{\omega }\cup \partial \Omega_{0}\cup \partial \Omega_{f}\;,\\ \;\omega =\{a\}\times (-\frac{1}{2}\pi ,0)\times (-\ell ,\ell )\;,\\ \partial \Omega_{0}=\{\{a_{0}\}\times (-\pi ,\pi ]\times [-L,L]\}\cup \{(a_{0},a]\times (-\pi ,\pi ]\\ \;\times \{\{-L\}\cup \{L\}\}\}\;,\\ \partial \Omega_{f}=\{\{a\}\times (-\pi ,\pi ]\times (-L,L)\}\setminus \bar{\omega }\;. \end{array}$$

Thus, we can express the boundary conditions of the foundation as

$$\begin{array}{l} \;u{|}_{\partial \Omega_{0}}=0\;(\text{zero}-\text{Dirichlet}),\\ \;[\partial_{3}u^{1}+\partial_{1}u^{3}]{|}_{\partial \Omega_{f}}=0\;(\text{zero}-\text{Robin}),\\ \;[r^{2}\partial_{3}u^{2}+\partial_{2}u^{3}]{|}_{\partial \Omega_{f}}=0\;(\text{zero}-\text{Robin}),\\ {}[\bar{\lambda }(\partial_{1}u^{1}+\partial_{2}u^{2}+r^{-1}u^{3})+(\bar{\lambda }+2\bar{\mu })\partial_{3}u^{3}]{|}_{\partial \Omega_{f}}=0(\text{zero}-\text{Robin}). \end{array}$$

Let $u{|}_{\omega }=(u^{1}(x,\theta ,a),u^{2}(x,\theta ,a),u^{3}(x,\theta ,a))$ be the displacement field of the shell-membrane. With some calculations, one can find the first fundamental form tensor to be $F_{\text{[I]}}=\mathrm{diag}\left(1,a^{2}\right)$ and the covariant derivatives to be

$$\begin{array}{l} \nabla_{\text{​}1}u^{1}=\partial_{1}u^{1}\;,\;\;\nabla_{\text{​}1}u^{2}=\partial_{1}u^{2}\;,\\ \nabla_{\text{​}2}u^{1}=\partial_{2}u^{1}\;,\;\;\nabla_{\text{​}2}u^{2}=\partial_{2}u^{2}\;. \end{array}$$

Considering the case $h^{2}\rho_{\alpha }^{\gamma }(u)\rho_{\gamma }^{\alpha }(u)\ll \epsilon_{\alpha }^{\gamma }(u)\epsilon_{\gamma }^{\alpha }(u)$, with some further calculations one can express the governing equations of the shell-membrane as:

If $[2\nu_{F}u^{3}+{\left(u^{1}u^{1}+r^{2}u^{2}u^{2}\right)}^{\frac{1}{2}}]{|}_{\omega }<0$, then

$$\begin{array}{l} (\Lambda +\mu )\partial^{1}(\nabla_{\text{​}\alpha }u^{\alpha })+\mu \Delta u^{1}+\frac{1}{a}\Lambda \partial^{1}u^{3}-\frac{1}{h}\text{Tr}(T_{3}^{1}(u))=0\;,\\ (\Lambda +\mu )\partial^{2}(\nabla_{\text{​}\alpha }u^{\alpha })+\mu \Delta u^{2}+\frac{1}{a}(\Lambda +2\mu )\partial^{2}u^{3}\\ \;-\frac{1}{h}\text{Tr}(T_{3}^{2}(u))=0\;,\\ \Lambda \partial_{1}u^{1}+(\Lambda +2\mu )\left(\partial_{2}u^{2}+\frac{1}{a}u^{3}\right)+\frac{a}{h}\text{Tr}(T_{3}^{3}(u))=0\;. \end{array}$$

If $[2\nu_{F}u^{3}+{\left(u^{1}u^{1}+r^{2}u^{2}u^{2}\right)}^{\frac{1}{2}}]{|}_{\omega }=0$, then

$$\begin{array}{l} (\Lambda +\mu )\partial^{1}(\nabla_{\text{​}\alpha }u^{\alpha })+\mu \Delta u^{1}-\frac{\Lambda }{2a\nu_{F}}\partial^{1}{\left(u^{1}u^{1}\text{​}+\text{​}a^{2}u^{2}u^{2}\right)}^{\frac{1}{2}}\\ \;-\frac{1}{h}\text{Tr}(T_{3}^{1}(u))-\frac{\left(\Lambda +\mu \right)}{4a\nu_{F}^{2}}u^{1}-\frac{\left(\bar{\lambda }+\bar{\mu }\right)}{4h\nu_{F}^{2}}u^{1}\\ +\frac{1}{2\nu_{F}}\frac{u^{1}}{{\left(u^{1}u^{1}\text{​}+\text{​}a^{2}u^{2}u^{2}\right)}^{\frac{1}{2}}}(\Lambda \partial_{1}u^{1}+(\Lambda +2\mu )\partial_{2}u^{2}\\ \;+\;\frac{a}{h}\left(\bar{\lambda }(\partial_{1}u^{1}+\partial_{2}u^{2})+\;(\bar{\lambda }+2\bar{\mu })\partial_{3}u^{3}\right))=0\;,\\ (\Lambda +\mu )\partial^{2}(\nabla_{\text{​}\alpha }u^{\alpha })+\mu \Delta u^{2}-\frac{\left(\Lambda +2\mu \right)}{2a\nu_{F}}\partial^{2}{\left(u^{1}u^{1}\text{​}+\text{​}a^{2}u^{2}u^{2}\right)}^{\frac{1}{2}}\\ \;-\frac{1}{h}\text{Tr}(T_{3}^{2}(u))-\frac{\left(\Lambda +\mu \right)}{4a\nu_{F}^{2}}u^{2}-\frac{\left(\bar{\lambda }+\bar{\mu }\right)}{4h\nu_{F}^{2}}u^{2}\\ +\;\frac{1}{2\nu_{F}}\frac{u^{2}}{{\left(u^{1}u^{1}\text{​}+\text{​}a^{2}u^{2}u^{2}\right)}^{\frac{1}{2}}}(\Lambda \partial_{1}u^{1}+(\Lambda +2\mu )\partial_{2}u^{2}\\ \;+\frac{a}{h}\left(\bar{\lambda }(\partial_{1}u^{1}+\partial_{2}u^{2})+\;(\bar{\lambda }+2\bar{\mu })\partial_{3}u^{3}\right))=0\;. \end{array}$$

The boundary of the shell-membrane can be decomposed into sub-boundaries as

$$\begin{array}{l} \;\partial \omega =\partial \omega_{T_{0}}\cup \partial \omega_{T_{\text{max}}}\cup \partial \omega_{f}\;,\\ \;\partial \omega_{T_{0}}=[-\ell ,\ell ]\times \left\{-\frac{1}{2}\pi \right\}\;,\\ \partial \omega_{T_{\text{max}}}=[-\ell ,\ell ]\times \{0\}\;,\\ \;\partial \omega_{f}=\{\{-\ell \}\cup \{\ell \}\}\cup \left(-\frac{1}{2}\pi ,0\right)\;. \end{array}$$

Thus, we can express the boundary conditions of the shell-membranes as

$$\begin{array}{l} {}[\Lambda \partial_{1}u^{1}+(\Lambda +2\mu )(\partial_{2}u^{2}+a^{-1}u^{3})]{|}_{\partial \omega_{T_{0}}}=\tau_{0}\;(\text{traction})\;,\\ {}[\Lambda \partial_{1}u^{1}+(\Lambda +2\mu )(\partial_{2}u^{2}+a^{-1}u^{3})]{|}_{\partial \omega_{T_{\text{max}}}}=\tau_{\text{max}}\;(\text{traction})\;,\\ \;[\partial_{2}u^{1}+a^{2}\partial_{1}u^{2}]{|}_{\partial \omega_{f}}=0\;(\text{zero}-\text{Robin})\;,\\ \;[(\Lambda +2\mu )\partial_{1}u^{1}+\Lambda (\partial_{2}u^{2}+a^{-1}u^{3})]{|}_{\partial \omega_{f}}=0\;(\text{zero}-\text{Robin})\;. \end{array}$$

A second-order accurate finite-difference method is again employed in conjunction with Newton's method for nonlinear systems. A modestly fine grid is chosen and the iterative process is terminated once $\left|1-||u_{m+1}|{|}_{\ell^{2}}/||u_{m}|{|}_{\ell^{2}}\right|$ falls below a certain value (10^−10^ in the calculations shown here). We attempt to model a stiff shell-membrane on a relatively flaccid foundation with a large coefficient of friction. To do so, we keep the values ν_*F*_ = 1, *h* = 0.001, *a* = 1, $\ell =\frac{1}{4}$, $L=\frac{1}{2}$, *E* = 10^3^, $\nu =\frac{1}{4}$, $\bar{\nu }=0$, τ_0_ = 1 and τ_max_ = 2 fixed for all experiments. Note that some preliminary work in chapter 4 of Jayawardana (2016) found that if (i) the overlying body is relatively thin, (ii) it has a relatively high Young's modulus, (iii) the coefficient of friction is high, and (iv) the mean curvature is a constant, then the solution for the shell model with friction is in relatively good agreement with a numerical model using Kikuchi and Oden's original friction law in chapter 10 of Kikuchi and Oden (1988), thus justifying the choice of our parameters.

**Figure 8:**
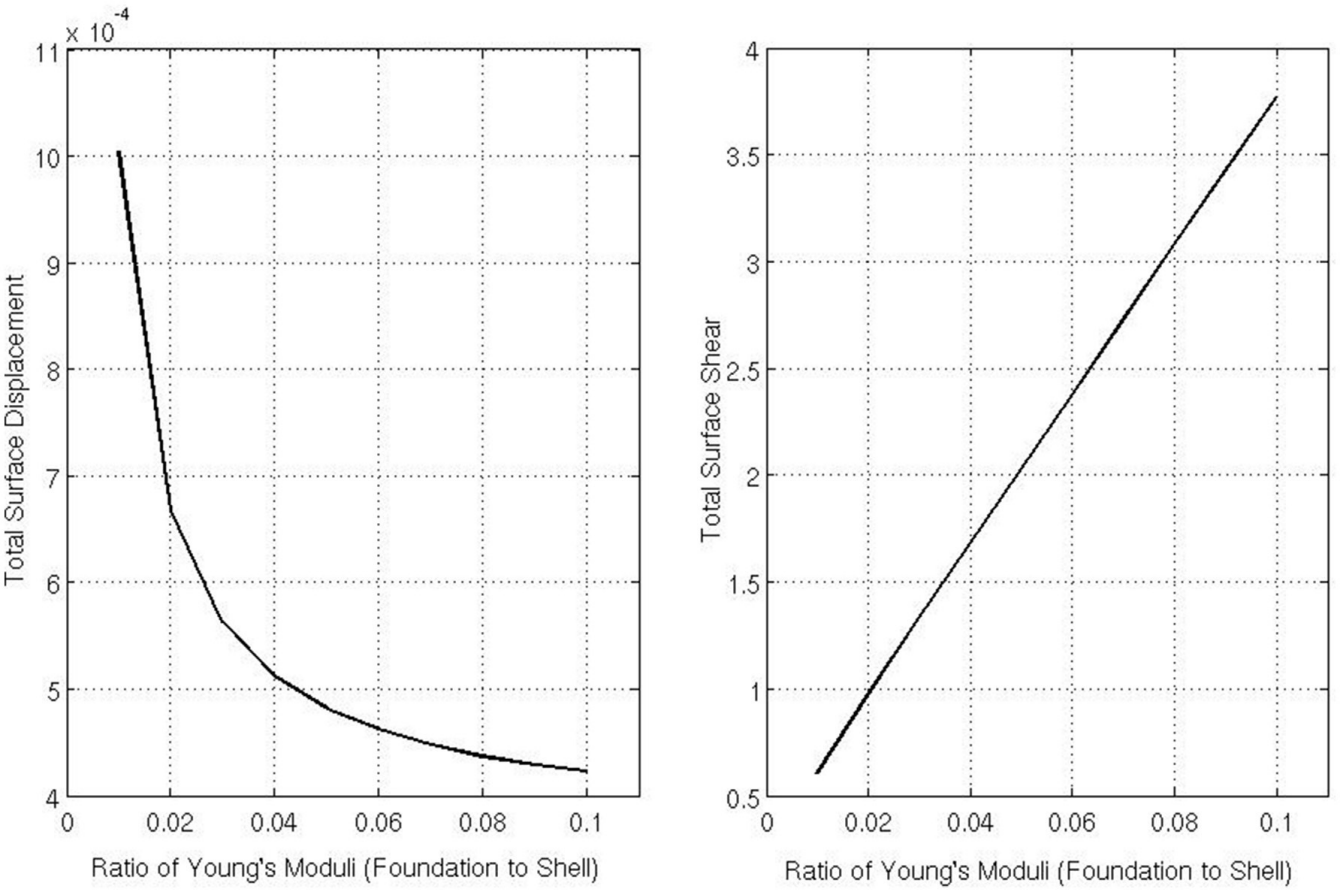
**Displacement and the shear in the contact region for varying Ē/***E*****.

Figure 8 shows the total surface displacement and the total surface shear at the outer boundary of the foundation for varying Ē (the Young's modulus of the foundation). The total displacement is calculated by $\left|u|{|}_{r=a}={\left(\sum_{\left\{\Delta x^{1},\Delta x^{2}\right\}}u_{i}u^{i}\right)}^{\frac{1}{2}}{|}_{r=a},\text{for }i\in \{1,2,3\right\}$ and the total shear is calculated by $\left|\sum_{\left\{\Delta x^{1},\Delta x^{2}\right\}}T_{\alpha }^{3}(u)T_{3}^{\alpha }(u){|}^{\frac{1}{2}}{|}_{r=a},\text{for }\alpha \in \{1,2\right\}$. For these simulations, we asserted that $a_{0}=\frac{1}{4}$ and *Ē* = {10, 20, 30, 40, 50, 60, 70, 80, 90, 100}. From Figure 8 one can see that as the Young's modulus of the foundation increases, the shear stress experienced on the underlying body surface increases, but the total displacement of the body decreases. Only the decrease in displacement, as the Young's modulus of the foundation increases, seems intuitive as when the Young's modulus increases, one would need to increase the amount of force applied to achieve the same amount of displacement. However, the increase in total surface shear is a more interesting result suggesting higher shear forces occur on the underlying surface as the body becomes stiffer despite the coefficient of friction remaining fixed.

**Figure 9:**
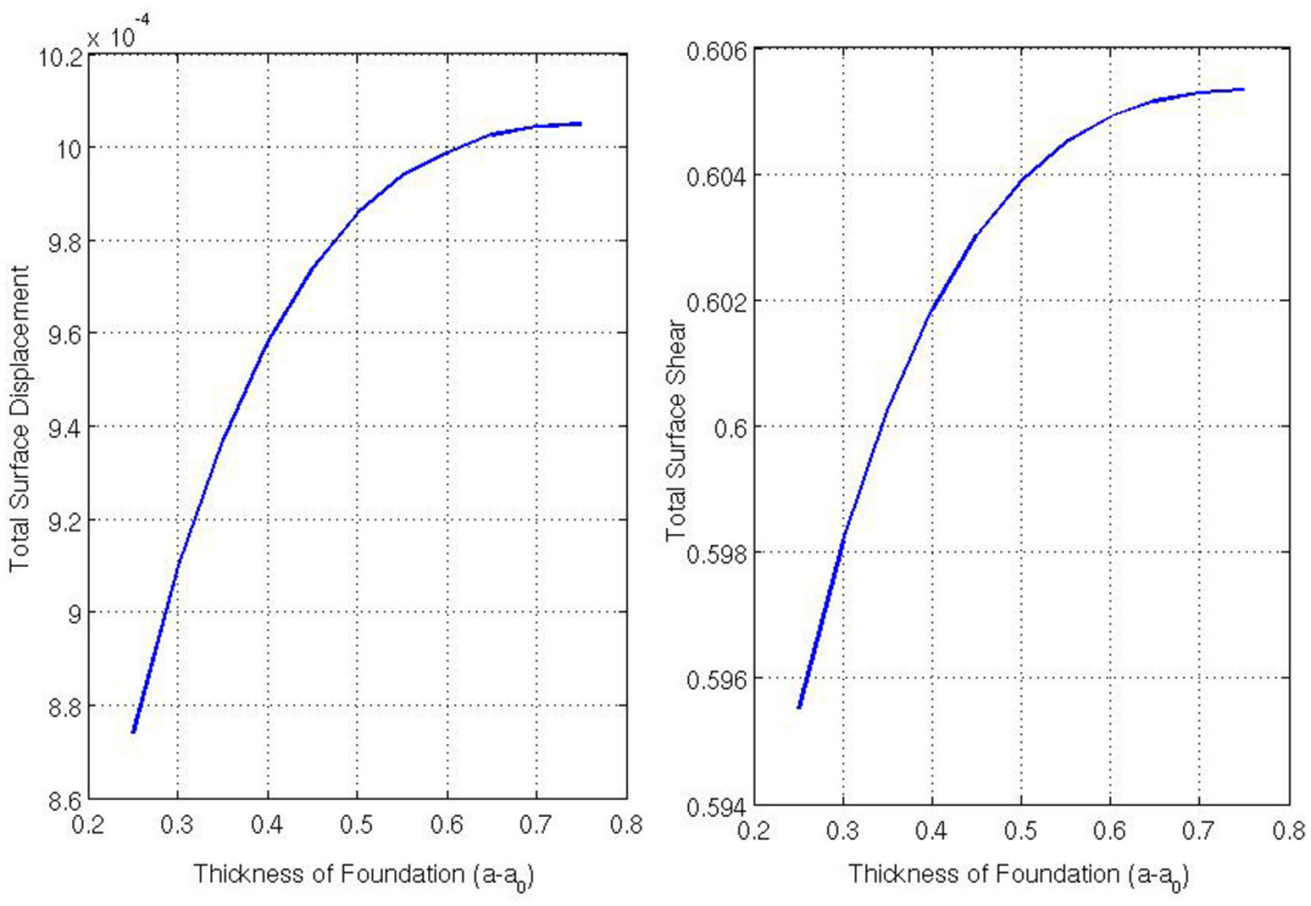
**Displacement and the shear in the contact region for varying foundation thickness**.

**Figure 10:**
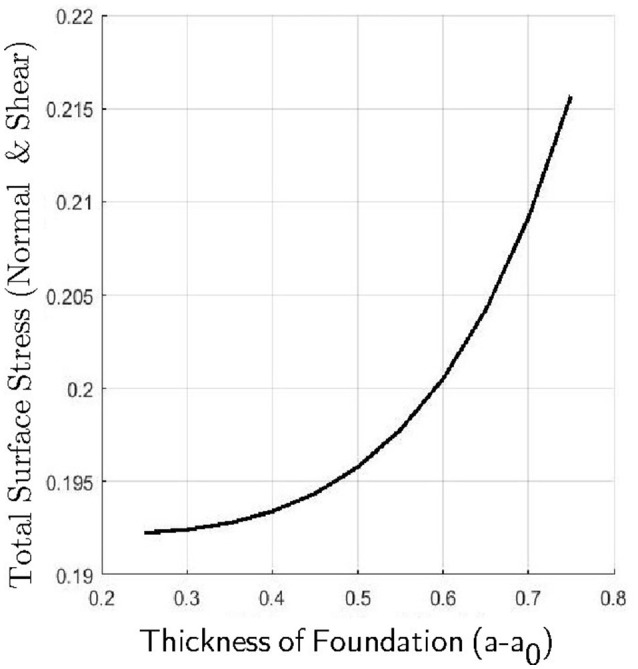
**Total surface stress (shear and normal) for varying foundation thickness**.

Figure 9 shows the total surface displacement and the total surface shear at the outer boundary of the foundation for varying *a*_0_. For these experiments, we asserted that $a_{0}\in \left\{\frac{5}{20},\frac{6}{20},\frac{7}{20},\frac{8}{20},\frac{9}{20},\frac{10}{20},\frac{11}{20},\frac{12}{20},\frac{13}{20},\frac{14}{20},\frac{15}{20}\right\}$ and *Ē* = 10. From Figure 9, one can see that as the thickness of the foundation increases, both the total displacement and the shearing stress on the surface of the body increases. The increase in displacement as the thickness of the foundation increases seems intuitive as there is more deformable matter available to be displaced but this appears again to lead to higher shear stress in the contact region. To explore this further, our final figure (Figure 10) shows the total surface stress (shear *and* normal stresses) at the outer boundary of the foundation. The total surface stress is calculated by $\left|\sum_{\left\{\Delta x^{1},\Delta x^{2}\right\}}T_{i}^{3}(u)T_{3}^{i}(u){|}^{\frac{1}{2}}{|}_{r=a},\text{where i}\in \{1,2,3\right\}$. These plots indicate that not only does a thicker foundation experience higher shear stresses but increasingly higher normal stresses with a consequent increase in the likelyhood of potential damage to the underlying body.

## 4. Conclusions

Two numerical models have been presented here that can provide insight into the forces generated by a compliant sheet of material under tension in contact with a rigid or deformable object. The aspects of geometry and deformability of the underlying body were examined separately by the mathematical models presented in Sections 2 and 3 respectively.

The model in Section 2 determines the coefficient of friction in the contact region between a thin membrane pulled dynamically at a constant speed and a rigid underlying body. The model of Kikuchi and Oden (1988) for Coulomb's law of static friction was extended to curvilinear coordinates, and a numerical model was used to investigate how the calculated coefficient of friction varies with different material and physical parameters. For parameters such as Poisson's ratio of the membrane, Young's modulus of the membrane and the speed of the membrane there was no significant variation in the determined coefficient of friction; this indicates that a capstan-type approach lacking dependence on these parameters should still produce accurate results. However, changes to the mass density of the fabric and the lateral (and thus Gaussian) curvature of the underlying body appear to lead to significant variations in the determined coefficient of friction which would not be captured by a capstan model. Even a remarkably small mass density of the fabric (which is assumed to be negligible in the capstan model) leads to the capstan model overestimating the actual coefficient of friction. This effect can be, to a certain extent, attributed to the fabric weight contributing to the overall tension and how this impacts via the logarithmic relation between the coefficient of friction and the tension ratio.

In varying the Gaussian curvature of the underlying body, the numerical model suggests that for a saddle-type geometry (as seen in real experiments such as Figure 2) the capstan approach may lead to a significant underestimate of the coefficient of friction. On the other hand, for a barrel-type geometry with positive Gaussian curvature the converse is true with the capstan equation potentially overestimating the coefficient of friction. The numerical model also indicates the intriguing possibility of an optimal barrel-type geometry where the coefficient of friction is minimized—a surprising result that certainly requires further investigation. We note, in passing, that the effect curvature appears to have on the coefficient of friction here possesses some similarities to how Kelvin's equation governs equilibrium vapor pressure over a curved surface (Skinner and Sambles, 1972; Fisher and Israelachvili, 1981) and how grain shape and size modify the rate of complex matter agglomeration (Gadomski and Rubı, 2003).

The model developed in Section 3 is for a thin shell-membrane under tension in frictional contact with an elastic foundation where static friction is imposed in the region of contact. The fact that our frictional law (e.g., Coulomb's law of static friction) must now be imposed on a *free boundary* because we no longer know *a priori* the location of the contact region significantly increases the computational complexity. To combat this, a modified, more computationally tractable, displacement-based static friction condition is derived from the model of Kikuchi and Oden (1988) for Coulomb's law of static friction in curvilinear coordinates. We can show that a set of governing equations for a two-body contact problem that incorporates this displacement-based static friction condition yields a unique solution. A numerical scheme for the two-body static friction contact problem is then developed where, this time, the coefficient of friction is specified. Using this model, we examine how the normal and tangential stresses and displacements computed by the model vary as we vary the stiffness and thickness of the underlying body. For a shell-membrane supported by an elastic foundation subjected to static friction we observe the following: (i) that as the Young's modulus of the elastic body increases, the magnitude of the displacement of the body surface decreases; (ii) that as the Young's modulus of the elastic body increases, the magnitude of the tangential shear stresses acting on the surface of body increases; (iii) that as the thickness of the elastic body increases, the magnitude of the surface displacements of the body increases; and (iv) that as the thickness of the elastic body increases, the magnitude of the tangential shear stresses acting on the body surface increases.

The shell-membrane in contact with a deformable elastic foundation model appears to indicate that both elastic and geometrical properties of the elastic foundation significantly affect the stress and deformation of the underlying tissue. This is reflected in the numerical results via a strong positive correlation between the thickness of the foundation and the amount of stress transferred from the shell-membrane to deforming the foundation. Indeed, with the applied tension and coefficient of friction fixed in the numerical model, the amount of stress experienced by the underlying elastic body appears to depend rather significantly on its geometry (thickness and curvature) and elastic properties, which are features that are neglected by capstan-type model approaches.

The models presented in this paper highlight some very interesting results and lead to a number of questions which should be pursued in terms of experimental design and to improve quantification of the frictional forces generated by nonwoven fabrics. One important issue is to examine how to measure the curvature of the contact region and designing experiments that can see how curvature affects the relationship between normal and tangential forces in the contact region. It would also be wise to test if the experimental results are indeed sensitive to variations in the mass density of the fabric. An extended capstan model that incorporates mass density is proposed in Jayawardana (2016) and other modifications to the capstan model could be considered. Experiments involving deformable underlying bodies with well-known material parameters would be useful to validate our second model and further our understanding about how experiments on quantifying frictional forces involving human subjects should deal with significant skin deformation, such as in the examples shown in Figure 2.

Some future work is planned for the models presented, including proving the existence of a unique solution in the model of Section 2 and validating the implementation of the model of Coulomb's law of friction in Kikuchi and Oden (1988) for dynamic friction. For the elastic foundation model (Section 3), further theoretical research on rigorously applying static and dynamic friction at a contact region which is a free boundary, on introducing finite deformations, and on how such a numerical model can be effectively used to provide insight from experimental data are under investigation. Finally, in terms of accurately modeling the response of the skin and underlying tissue, some consideration should be given to exploring beyond Coulomb's law of friction to incorporate the adhesive and repulsive intermolecular forces (such as via hydration) occurring across a variety of spatial scales, leading to Derjaguin-type frictional laws (Gadomski, 2015).

## Ethics statement

This paper is fundamentally a mathematical paper but there is one figure (Figure 2) containing images of experiments on human subjects from a previous project that is shown as motivation for the mathematical modeling work. The images show deformation in the skin and underlying soft tissue generated during measurements of friction between strips of nonwoven fabric and the volar forearms of female volunteers. The work was conducted with the approval of London Stanmore Research Ethics Committee and The Whittington Hospital NHS R&D office, September 2011. It was carried out in accordance with the recommendations of IRAS (Integrated Research Application System) Help guidance notes for HRA approval (Health Research Authority) with written informed consent from all subjects. All subjects gave written informed consent in accordance with the Declaration of Helsinki. Investigators interacting with elderly and disabled participants underwent DBS checks prior to REC submission and as a requirement for study approval.

## Author contributions

Research Design: KJ, NO, and AC. Mathematical Modeling and Analysis: KJ and NO. Clincial data analysis and guidance: AC. KJ, NO, and AC all contributed to the writing of the manuscript.

## Funding

This work was supported by The Dunhill Medical Trust [grant number R204/0511]; UCL Impact Studentship.

### Conflict of interest statement

The authors declare that the research was conducted in the absence of any commercial or financial relationships that could be construed as a potential conflict of interest.
